# The Penicillin-Binding Protein PbpP Is a Sensor of β-Lactams and Is Required for Activation of the Extracytoplasmic Function σ Factor σ^P^ in Bacillus thuringiensis

**DOI:** 10.1128/mBio.00179-21

**Published:** 2021-03-23

**Authors:** Kelsie M. Nauta, Theresa D. Ho, Craig D. Ellermeier

**Affiliations:** a Department of Microbiology and Immunology, Carver College of Medicine, University of Iowa, Iowa City, Iowa, USA; b Graduate Program in Genetics, University of Iowa, Iowa City, Iowa, USA; Massachusetts Institute of Technology

**Keywords:** σ factors, cell envelope, stress response, signal transduction, regulation of gene expression, sigma factors

## Abstract

β-Lactams are a class of antibiotics that target the synthesis of peptidoglycan, an essential component of the cell wall. β-Lactams inhibit the function of penicillin-binding proteins (PBPs), which form the cross-links between strands of peptidoglycan. Resistance to β-lactams complicates the treatment of bacterial infections. In recent years, the spread of β-lactam resistance has increased with growing intensity. Resistance is often conferred by β-lactamases, which inactivate β-lactams, or the expression of alternative β-lactam-resistant PBPs. σ^P^ is an extracytoplasmic function (ECF) σ factor that controls β-lactam resistance in the species Bacillus thuringiensis, Bacillus cereus, and Bacillus anthracis. σ^P^ is normally held inactive by the anti-σ factor RsiP. σ^P^ is activated by β-lactams that trigger the proteolytic destruction of RsiP. Here, we identify the penicillin-binding protein PbpP and demonstrate its essential role in the activation of σ^P^. Our data show that PbpP is required for σ^P^ activation and RsiP degradation. Our data suggest that PbpP acts as a β-lactam sensor since the binding of a subset of β-lactams to PbpP is required for σ^P^ activation. We find that PbpP likely directly or indirectly controls site 1 cleavage of RsiP, which results in the degradation of RsiP and, thus, σ^P^ activation. σ^P^ activation results in increased expression of β-lactamases and, thus, increased β-lactam resistance. This work is the first report of a PBP acting as a sensor for β-lactams and controlling the activation of an ECF σ factor.

## Abstract (precis)

The bacterial cell envelope is the target for numerous antibiotics. Many antibiotics target the synthesis of peptidoglycan, which is a central metabolic pathway essential for bacterial survival.

## INTRODUCTION

The bacterial cell wall is essential for cell viability under most environmental conditions. Peptidoglycan is the major component of the cell wall and is responsible for maintaining cell shape, preventing lysis under turgor pressure, and protecting the cell from extracellular stresses. Peptidoglycan is composed of chains of repeating *N*-acetylglucosamine (NAG) and *N*-acetylmuramic acid (NAM) subunits that are cross-linked by pentapeptide side chains extending from the NAM subunits (1, 2). In Gram-positive organisms, the peptidoglycan forms a multilayer matrix that encases the plasma membrane (3).

Penicillin-binding proteins (PBPs) are some of the enzymes responsible for peptidoglycan synthesis. In the cytosol, dimers of NAG-NAM with pentapeptide side chains are synthesized and then flipped outside the cell membrane. These dimers are added to the growing peptidoglycan polymer by transglycosylation, which results in strands of repeating NAG-NAM subunits. These strands are cross-linked by transpeptidation of their pentapeptide side chains in a reaction carried out by PBPs. There are two types of high-molecular-weight PBPs. Type a PBPs have both transglycosylase activity and transpeptidase activity. Type b PBPs have only transpeptidase activity but work in concert with monofunctional SEDS (shape, elongation, division, sporulation) family transglycosylases to synthesize peptidoglycan (2, 4). The activities of type a PBPs and type b PBPs are required for cell viability (5–7).

β-Lactam and cephalosporin antibiotics inhibit peptidoglycan synthesis by forming a covalent bond with the transpeptidase active-site serine of PBPs (5, 8, 9). This inhibition prevents cross-linking of the peptide side chains, which results in peptidoglycan instability and lysis during cell growth (10). Resistance to β-lactams and cephalosporins is a growing problem that complicates the treatment of bacterial infections. Resistance to β-lactams is usually due to the secretion of β-lactamases, which destroy the antibiotic by cleaving the β-lactam ring, or mutations that lead to modification of the transpeptidase active sites of PBPs and prevent β-lactam binding (11, 12).

In response to stresses like antimicrobial peptides or antibiotics, many bacteria utilize alternative σ factors to regulate subsets of genes required for the stress response. The extracytoplasmic function (ECF) σ factor family is the largest and most diverse group of alternative σ factors and represents the “third pillar” of bacterial signal transduction (13–15). ECF σ factors are part of the σ^70^ family but contain only region 2 and region 4.2 of σ^70^. These regions bind to the −10 and −35 regions of promoters, respectively (13, 16). Many ECF σ factors are held inactive by anti-σ factors (13, 17, 18). The activation of these ECF σ factors requires release from their cognate anti-σ factors to allow the transcription of specific stress response genes.

A recent study identified >150 different families of ECF σ factors (15). The roles of the vast majority of these σ factors remain poorly understood; however, of the studied ECF σ factors, the mechanisms of ECF σ factor activation are diverse (18–20). One common mechanism known to control ECF σ factor activation is the proteolytic destruction of the anti-σ factor (18, 21). Among those ECF σ factor systems that use proteolytic destruction of the anti-σ factor, the mechanisms controlling the initiation of this proteolytic cascade are diverse (21). In Escherichia coli, the activation of σ^E^ is controlled by the binding of misfolded outer membrane proteins to the site 1 protease DegS and lipopolysaccharide (LPS) binding to RseB (a negative regulator of σ^E^ activation) (22–25). These binding events lead to the cleavage of the anti-σ factor RseA at site 1 by DegS (26). In Bacillus subtilis, the activation of σ^V^ by lysozyme is controlled by the direct binding of the anti-σ factor RsiV to lysozyme and then cleavage of RsiV at site 1 by signal peptidase (27–32).

In Bacillus anthracis, Bacillus cereus, and Bacillus thuringiensis, resistance to penicillin and other β-lactam antibiotics is dependent upon σ^P^, an ECF σ factor (33, 34). σ^P^ was originally classified as a member of the ECF01 group of ECF σ factors but was recently reclassified to the ECF265 group, the members of which are primarily found in *Firmicutes* (14, 15). Approximately 50% of ECF265 σ factors are associated with an anti-sigma factor that contains a single transmembrane helix (15). Little is known about how the activity of the ECF265 σ group is controlled, and σ^P^ could represent a model to understand the activation of this subclass of ECF σ factors.

**FIG 1:**
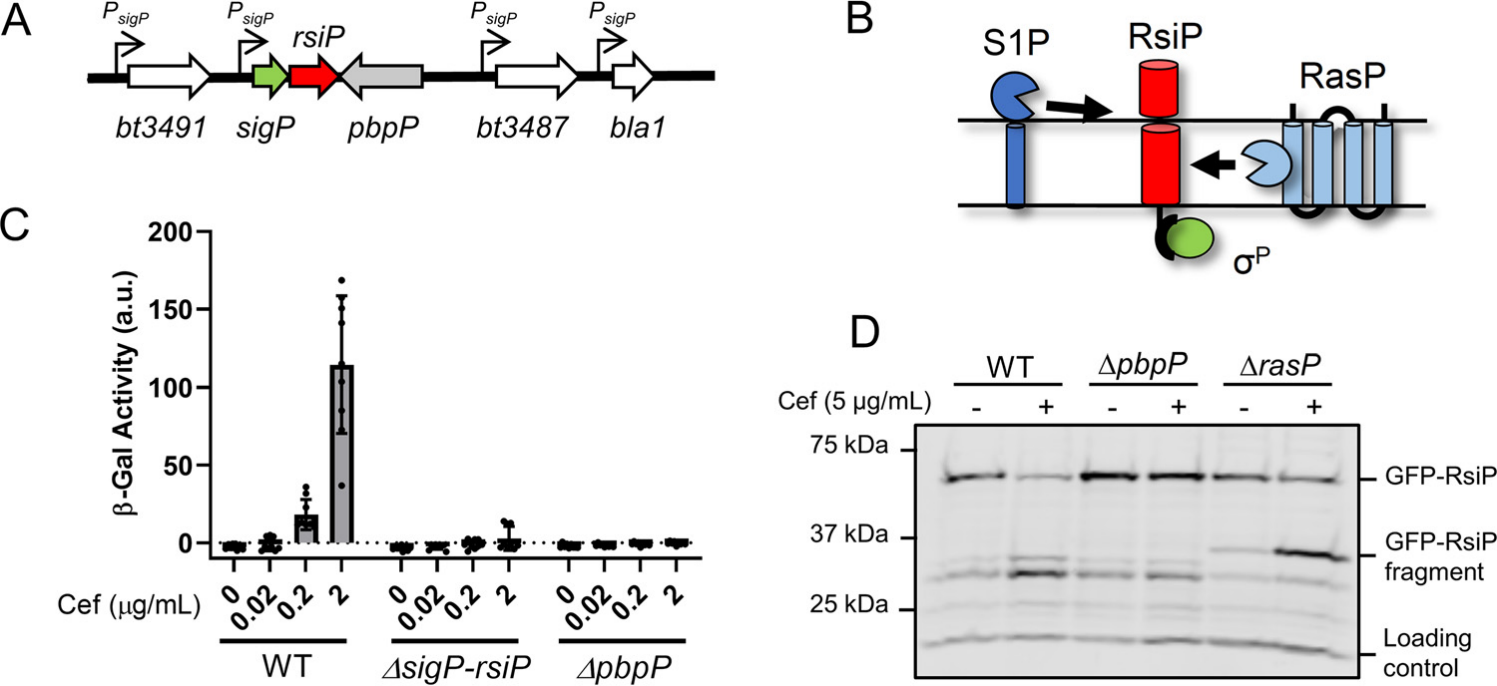
PbpP is required for σ^P^ activation. (A) PbpP (HD73_3488) (gray) is encoded immediately downstream of *sigP* (green) and *rsiP* (red) in B. thuringiensis. (B) Model of σ^P^ activation. The anti-σ factor RsiP (red) sequesters σ^P^ (green) in the absence of β-lactams. In the presence of β-lactams, RsiP is sequentially cleaved by an unknown site 1 protease (S1P) (dark blue) and RasP (light blue) (34). (C) PbpP is required for the activation of σ^P^. All strains contain the reporter *P_sigP_-lacZ*. The relevant genotypes of the tested strains included WT (THE2549), Δ*sigP-rsiP* (EBT232), and Δ*pbpP* (EBT151). Cells were grown to mid-log phase (OD of 1.0 to 1.4), washed, and resuspended in LB medium and LB medium plus cefoxitin (Cef) (0.02 to 2 μg/ml). β-Galactosidase (β-Gal) activities were calculated as described in Materials and Methods. Experiments were performed in technical and biological triplicate, and standard deviations are represented by error bars. a.u., arbitrary units. (D) PbpP is required for cefoxitin-induced degradation of RsiP. All strains contain the plasmid pBT13 (*P_tet_-gfp-rsiP*) and the following relevant genotypes: WT (THE360), Δ*pbpP* (EBT512), and Δ*rasP* (EBT366). The strains were grown to mid-log phase at 37°C in ATc (100 ng/ml), concentrated, and resuspended in 100 μl of LB medium or LB medium with cefoxitin (5 μg/ml) for 1 h. Immunoblotting was performed using anti-GFP antisera. Streptavidin IR680LT was used to detect AccB (HD73_4487), which served as a loading control (51, 52). A color blot showing both anti-GFP and streptavidin on a single gel is shown in Fig. S2 in the supplemental material. Numbers at the left indicate molecular masses of the ladder in kilodaltons.

σ^P^ activity is inhibited by the anti-σ factor RsiP, which contains a single transmembrane helix. The activation of σ^P^ results in the expression of at least two genes that encode β-lactamases and are involved in resistance to penicillin, ampicillin, and other β-lactam antibiotics. σ^P^ also activates the expression of its operon, thus controlling the expression of *sigP* and *rsiP* (33, 34). We previously demonstrated that σ^P^ is activated in the presence of a subset of β-lactams, ampicillin, methicillin, cefoxitin, cephalothin, and cefmetazole, but not other cell envelope stresses (34). We also identified a subset of β-lactams that do not activate σ^P^: piperacillin, cefsulodin, and cefoperazone (34). In response to the activating β-lactams, RsiP is destroyed by a cascade of proteases, resulting in σ^P^ activation (34). An unidentified site 1 protease initiates the proteolytic cascade by cleaving RsiP at site 1, which is then followed by cleavage at site 2 by RasP, the highly conserved site 2 protease (34) (Fig. 1B). Here, we demonstrate that β-lactam activation of σ^P^ is dependent on the PBP HD73_3488 (also known as HD73_RS17405), which we have named PbpP. Our data indicate that PbpP is required for site 1 cleavage of RsiP in response to β-lactams, but PbpP is likely not the site 1 protease. Our data suggest that PbpP likely functions as a sensor of β-lactams by directly binding β-lactams and triggering σ^P^ activation by promoting site 1 cleavage of RsiP.

10.1128/mBio.00179-21.2 FIG S2 PbpP is required for cefoxitin-induced degradation of RsiP (color version of Fig. 1D). (A) All strains contain the plasmid pBT13 (*P_tet_-gfp-rsiP*) and the following relevant genotypes: WT (THE360), Δ*pbpP* (EBT512), and Δ*rasP* (EBT366). The strains were grown to mid-log phase at 37°C in ATc (100 ng/ml), concentrated, and resuspended in 100 μl of LB medium or LB medium with cefoxitin (5 μg/ml) for 1 h. Immunoblotting was performed as described in Materials and Methods using antisera against GFP. Streptavidin IR680LT was used to detect AccB (HD73_4487), which served as a loading control (51, 52). A color blot showing both anti-GFP and streptavidin on a single gel is shown. Numbers at the right indicate molecular masses of the ladder in kilodaltons. Download FIG S2, PDF file, 0.2 MB. Copyright © 2021 Nauta et al. 2021 Nauta et al. https://creativecommons.org/licenses/by/4.0/ This content is distributed under the terms of the Creative Commons Attribution 4.0 International license.

## RESULTS

### PbpP is required for σ^P^ activation.

Bacillus thuringiensis, B. cereus, and B. anthracis contain two open reading frames in the *sigP* region that encode predicted penicillin-binding proteins (PBPs). In Bacillus thuringiensis subsp. *kurstaki* HD73, these PBPs are called *pbpP* (HD73_3488) and *bt3491* (HD73_3491). We also identified a third open reading frame that appears to be found only in Bacillus thuringiensis subsp. *kurstaki* HD73, called *bt3487* (*HD73_3487*) (Fig. 1A). Although they are not located in the same operon as *sigP* and *rsiP*, we hypothesized that they may play a role in the response of σ^P^ to β-lactams because PBPs have been well characterized as targets of β-lactam antibiotics (9, 35). Additionally, genes involved in the same signaling system are often located in the neighboring regions. To determine if BT3487, PbpP, and BT3491 were required for the response of σ^P^ to β-lactams, we generated strains with in-frame deletions of each of the genes and measured the effect on ampicillin resistance. We found that the deletion of *pbpP* led to a dramatic decrease in the ampicillin MIC similar to that of a Δ*sigP* mutant (Table 1) (33, 34). In contrast, strains with deletions in *bt3487* and *bt3491* had no effect on ampicillin resistance (not shown). We also determined that a Δ*pbpP* mutant is more sensitive to cefoxitin and cefmetazole than the wild type (WT) (Table 1).

**TABLE 1:** MICs of β-lactams

β-Lactam	Mean MIC (μg/ml) for strain ± SD^*a*^			Fold difference	
	WT	Δ*sigP*-*rsiP*	Δ*pbpP*	WT/Δ*sigP*-*rsiP*	WT/Δ*pbpP*
Ampicillin	16,000 ± 6,000	0.13 ± 0.09	3.2 ± 0.57	120,000	5,000
Cefoxitin	50.0 ± 0	7.8 ± 2.5	14 ± 5.9	6.4	3.6
Cefmetazole	11 ± 2.6	10.0 ± 4.2	4.7 ± 0.79	1.1	2.3
Cefsulodin	400 ± 0	200 ± 0	300 ± 120	2	1.3

a Experiments were performed in biological and technical triplicate.

We noted that a Δ*sigP*-*rsiP* mutant is more sensitive to β-lactams than a Δ*pbpP* mutant. We hypothesized that a Δ*pbpP* mutant may block σ^P^ activation in response to β-lactams but retains a basal level of σ^P^ activation that allows a low level of resistance to β-lactams. To monitor σ^P^ activity, we took advantage of the fact that σ^P^ is required for the transcription of its promoter (*P_sigP_*); thus, we inserted a *P_sigP_-lacZ* promoter fusion into the *thrC* locus (33, 34). To determine if PbpP played a role in σ^P^ activation, we tested the effect of a *pbpP* deletion on σ^P^ activity by monitoring *P_sigP_-lacZ* expression. Interestingly, we did not observe activation of σ^P^ in the Δ*pbpP* mutant in the presence of cefoxitin (see Fig. S1A in the supplemental material). We complemented the Δ*pbpP* mutant with *pbpP^+^* on a plasmid under the control of its native promoter. We found that σ^P^ was activated in the presence of cefoxitin to an extent similar to that observed for the WT (Fig. S1A). To reinforce our finding that Δ*pbpP* results in the loss of σ^P^ activation, we conducted β-galactosidase assays to quantify the effect on σ^P^ activation. As previously reported, *P_sigP_-lacZ* expression is induced in a dose-dependent manner in response to increased cefoxitin concentrations in the WT (Fig. 1C) (34). Consistent with previous observations, we did not observe induction of *P_sigP_-lacZ* in the Δ*sigP*-*rsiP* mutant because σ^P^ is required for transcription from *P_sigP_* (34). We found that the deletion of *pbpP* resulted in the loss of *P_sigP_-lacZ* expression at every concentration tested (Fig. 1C). Taken together, our data suggest that PbpP is required for the activation of σ^P^, thereby altering the transcription of the σ^P^ regulon and β-lactam resistance.

10.1128/mBio.00179-21.1 FIG S1 σ^P^ is not activated in the absence of PbpP, and *P_pbpP_* is not induced by *sigP*. (A) *sigP* is not activated in the Δ*pbpP* mutant. All strains contain the P*_sigP_-lacZ* reporter and either the empty vector (EV) (pAH9) WT (EBT169), Δ*sigP-rsiP* (EBT251), Δ*pbpP* (EBT344), pEBT10 (pAH9-*pbpP*^+^) WT (EBT274), Δ*sigP-rsiP* (EBT275), or Δ*pbpP* (EBT276) genotype. Strains were grown to mid-log phase and spotted on LB agar containing X-Gal (50 μg/ml) with and without cefoxitin (5 μg/ml). (B) *P_pbpP_* is not activated by cefoxitin. *P_sigP_*-*lacZ* (THE2549) and *P_pbpP_*-*lacZ* (EBT234) were grown to mid-log phase at 37°C and spotted on LB agar containing 100 μg/ml X-Gal with or without cefoxitin (5 μg/ml). The plates were incubated overnight at 30°C. (C) *P_pbpP_*-*lacZ* is not activated by cefoxitin. Cultures of three strains, the parent (AW43 no reporter), *P_sigP_-lacZ* (THE2549), and *P_pbpP_-lacZ* (EBT234) strains, were grown to mid-log phase, cefoxitin was added, the cultures were incubated for 1 h, and β-galactosidase activity was measured. This experiment was done in technical and biological triplicate, and standard deviations are represented by error bars. Download FIG S1, PDF file, 0.2 MB. Copyright © 2021 Nauta et al. 2021 Nauta et al. https://creativecommons.org/licenses/by/4.0/ This content is distributed under the terms of the Creative Commons Attribution 4.0 International license.

### PbpP is required for site 1 cleavage of RsiP.

Because our data suggest that PbpP is required for σ^P^ activation, we hypothesized that PbpP is required for RsiP degradation. To test this, we compared the effects of cefoxitin on the degradation of green fluorescent protein (GFP)-RsiP in WT, Δ*pbpP*, and Δ*rasP* mutant strains. We previously showed that GFP-RsiP is functional and localized to the membrane (34). We found that the levels of full-length GFP-RsiP decreased in the WT in the presence of cefoxitin (Fig. 1D) (34). When a Δ*rasP* mutant, which lacks the site 2 protease, was incubated with cefoxitin, we observed a decrease in full-length GFP-RsiP and the buildup of an intermediate GFP-RsiP fragment, indicating the loss of site 2 cleavage (Fig. 1D) (34). This GFP-RsiP fragment is approximately the predicted size for a site 1 protease cleavage product. In contrast, we found that full-length GFP-RsiP levels did not decrease in the Δ*pbpP* mutant when grown in the presence of cefoxitin (Fig. 1D). This suggests that PbpP is required for site 1 cleavage of RsiP and, thus, σ^P^ activation.

### PbpP is a penicillin-binding protein.

**FIG 2:**
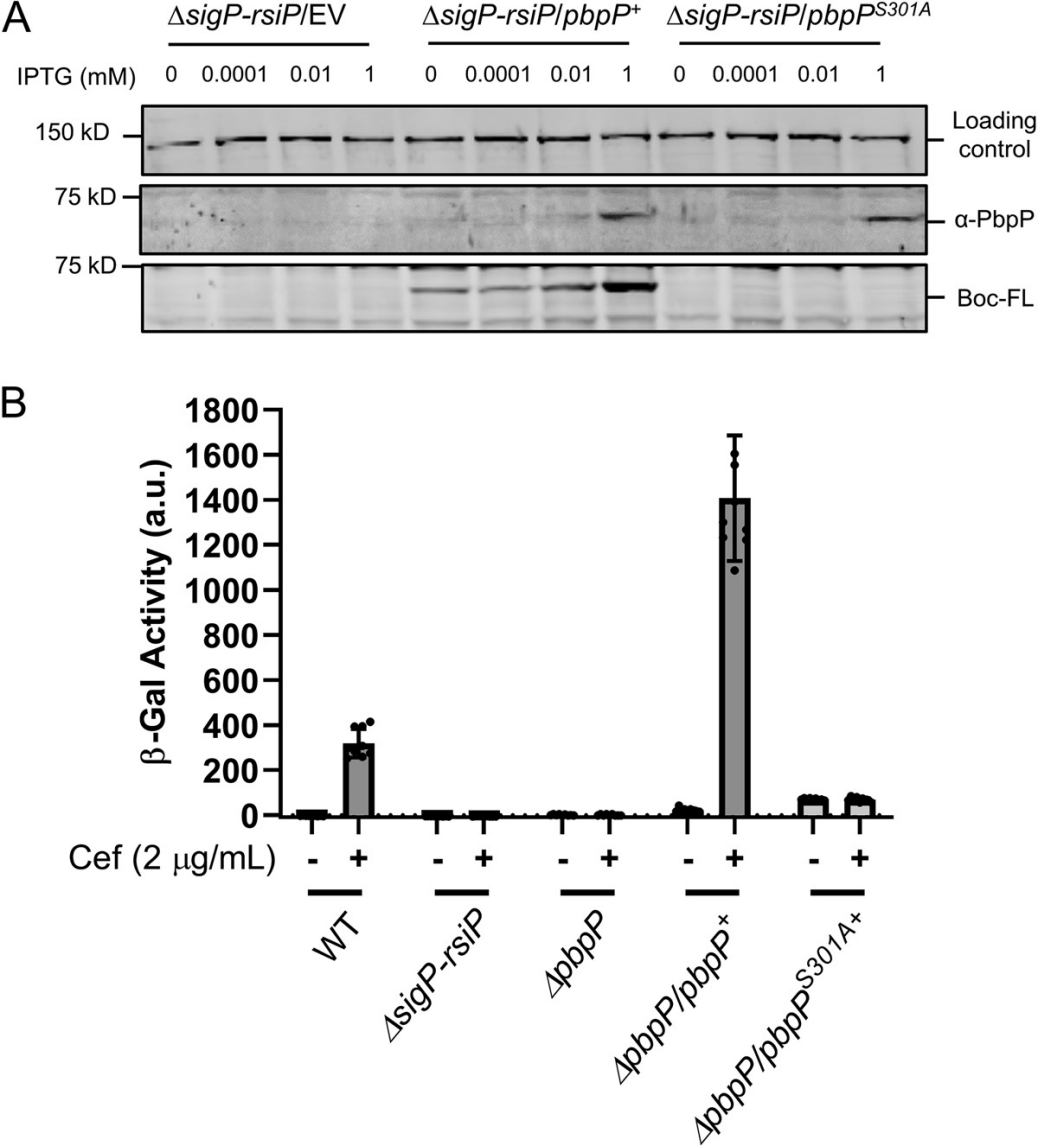
PbpP is a penicillin-binding protein. (A) S301 is the active-site serine of PBP. All strains contain Δ*sigP-rsiP* and either the empty vector (EV) (CDE3214), *P_IPTG_*-*pbpP*^+^ (CDE3248), or *P_IPTG_*-*pbpP^S301A^* (CDE3243). Cells were grown to mid-log phase with various concentrations of IPTG. Cells were concentrated, resuspended, and incubated with Bocillin-FL (50 μg/ml). The proteins were then separated by SDS-PAGE, immunoblotting was performed using anti-PbpP antisera and Bocillin-FL, and streptavidin IR680LT was used to detect HD73_4231 (PycA homolog), which served as a loading control (51, 52). Figure S3A in the supplemental material is the color blot showing anti-PbpP antisera, Bocillin-FL, and streptavidin in a single image. (B) *pbpP^S301A^* phenocopies Δ*pbpP.* All strains contain the reporter *P_sigP_-lacZ* and were of the following genotypes: WT (THE2549), Δ*sigP-rsiP* (EBT232), Δ*pbpP* (EBT151), Δ*pbpP* ICE*Bs1*::*pbpP^+^* (EBT773), and Δ*pbpP* ICE*Bs1*::*pbpP^S301A^* (EBT772). The strains were grown to mid-log phase and incubated without or with cefoxitin for 1 h, and β-galactosidase activity was measured. Experiments were performed in technical and biological triplicate, and standard deviations are represented by error bars.

A defining feature of PBPs is the ability to covalently bind β-lactams (9, 36). We sought to determine if PbpP has the capacity to bind β-lactams. We tested if PbpP could bind Bocillin-FL (Boc-FL), a fluorescent β-lactam consisting of penicillin V and BODIPY FL dye (37). We found that Bocillin-FL was degraded when σ^P^ was activated (Fig. S3B). In a Δ*sigP*-*rsiP* mutant, we found that Bocillin-FL was not degraded, suggesting that σ^P^-regulated β-lactamases are likely responsible for Bocillin-FL degradation (Fig. S3B). To perform Bocillin-FL labeling experiments, we expressed *pbpP* from an isopropyl-β-d-thiogalactopyranoside (IPTG)-inducible promoter in a Δ*sigP*-*rsiP* mutant. We labeled cells with Bocillin-FL and blotted them with anti-PbpP antisera (37). We observed a fluorescent band at approximately 66 kDa with both Bocillin-FL and anti-PbpP antisera. This band was the predicted size of PbpP; it increased in intensity with increasing IPTG concentrations and was not observed in the empty vector (EV) control (Fig. 2A and Fig. S3A). This demonstrates that PbpP binds β-lactams. We also noted that the lack of a fluorescent band corresponding to PbpP in the EV suggests that the levels of PbpP in wild-type cells are not high enough to be detected by Bocillin-FL labeling.

10.1128/mBio.00179-21.3 FIG S3 S301 is the active-site serine (color version of Fig. 2A). (A) S301 is the active-site serine of PBP. All strains contain Δ*sigP-rsiP* and either the empty vector (EV) (CDE3214), *P_IPTG_*-*pbpP*^+^ (CDE3248), or *P_IPTG_*-*pbpP^S301A^* (CDE3243). Cells were grown to mid-log phase with various concentrations of IPTG. Cells were concentrated, resuspended, and incubated with Bocillin-FL (50 μg/ml). The proteins were then separated by SDS-PAGE, and immunoblotting was performed as described in Materials and Methods using antisera against PbpP. Streptavidin IR680LT was used to detect HD73_4231 (PycA homolog), which served as a loading control (51, 52). (Top) Color blot showing anti-PbpP, Bocillin, and streptavidin on a single image. (Middle) Color blot showing anti-PbpP and streptavidin. (Bottom) Color blot showing Bocillin-FL alone. (B) Activation of σ^P^ causes degradation of Bocillin-FL. The relevant strains of the WT (THE2549), Δ*sigP-rsiP* (EBT232), and *rsiP^1–80^* (THE2628) genotypes were grown to mid-log phase and incubated with Bocillin-FL (50 μg/ml). Download FIG S3, PDF file, 0.3 MB. Copyright © 2021 Nauta et al. 2021 Nauta et al. https://creativecommons.org/licenses/by/4.0/ This content is distributed under the terms of the Creative Commons Attribution 4.0 International license.

All PBPs have an active-site serine that is acylated by β-lactams (36). We identified serine 301 (S301) as the likely active-site residue required for transpeptidation based on homology to other PBPs. To determine if S301 is the active-site serine, we mutated it to an alanine by site-directed mutagenesis and expressed *pbpP^S301A^* under the control of an IPTG-inducible promoter. In the strain producing PbpP^S301A^, the 66-kDa band was lost when imaging for Bocillin-FL (Fig. 2A and Fig. S3A). However, immunoblotting using anti-PbpP antisera detected a 66-kDa band corresponding to PbpP^S301A^, which is produced at levels similar to those of the WT protein (Fig. 2A; Fig. S3A). Thus, PbpP^S301A^ cannot covalently bind Bocillin-FL. This suggests that PbpP is a penicillin-binding protein, S301 is required for binding β-lactams, and S301 is likely the active-site serine.

### β-Lactam binding by PbpP is required for β-lactam-dependent activation of σ^P^.

We sought to determine if β-lactam binding to PbpP was required for σ^P^ activation using a PbpP^S301A^ active-site mutant. We complemented the Δ*pbpP* mutant with *pbpP^+^* and *pbp^S301A^* under the control of their native promoter in a single copy by integrating constructs at the B. subtilis integrative conjugative element (ICE*Bs1*) site in the B. thuringiensis chromosome (38). We found that PbpP*^+^* restored P*_sigP_-lacZ* expression in the presence of cefoxitin (Fig. 2B). In contrast, when we complemented the strain with *pbpP^S301A^*, we observed no increase in P*_sigP_-lacZ* expression in the presence of cefoxitin (Fig. 2B). These data suggest that binding of PbpP to β-lactams is required for β-lactams to activate σ^P^.

### Overexpression of *pbpP* and *pbpP^S301A^* leads to activation of σ^P^.

**FIG 3:**
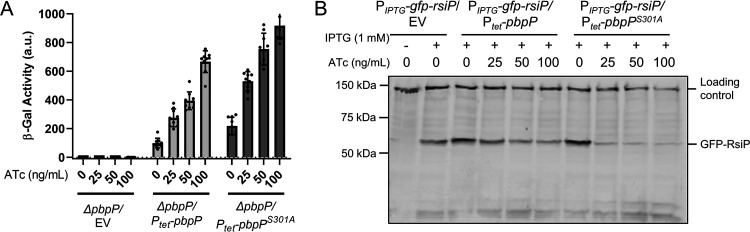
Overexpression of *pbpP* and *pbpP^S301A^* activates σ^P^. (A) Overexpression of *pbpP* and *pbpP^S301A^* results in activation of σ^P^. All strains contain the reporter *P_sigP_-lacZ* plus the following relevant genotypes: Δ*pbpP*/EV (EBT344), Δ*pbpP*/*P_tet_-pbpP^+^* (EBT327), and Δ*pbpP*/*P_tet_*-*pbpP^S301A^* (EBT1145). The cultures were grown to mid-log phase and incubated with anhydrotetracycline (ATc), and β-galactosidase activity was measured. This experiment was done in technical and biological triplicate, and standard deviations are represented by error bars. (B) Overexpression of *pbpP* causes degradation of RsiP. All strains harbor IPTG-inducible *gfp*-*rsiP* (*P_IPTG_-gfp-rsiP*) integrated at the ICE*Bs1* site (38) plus either the empty vector (EV) (pAH9) (EBT744), *P_tet_-pbpP^+^* (EBT742), or *P_tet_-pbpP^S301A^* (EBT1144). Strains were grown to mid-log phase with 1 mM IPTG and increasing concentrations of ATc. The cells were concentrated, resuspended in sample buffer, and separated by SDS-PAGE. The immunoblot was probed with anti-GFP antisera. Streptavidin IR680LT was used to detect HD73_4231 (PycA homolog), which served as a loading control (51, 52). A color blot showing both anti-GFP and streptavidin on a single gel is shown in Fig. S4A in the supplemental material.

We noted that the basal level of P*_sigP_-lacZ* expression was higher in the strains complemented with *pbpP^+^* and *pbp^S301A^* integrated at ICE*Bs1* than in WT B. thuringiensis (Fig. 2B). We reasoned that this might be due to higher basal levels of expression of *pbpP* and *pbpP^S301A^* at the ICE*Bs1* site. Thus, we sought to determine the effect of the overexpression of *pbpP^+^* and *pbpP^S301A^* on σ^P^ activation. We expressed *pbpP^+^* or *pbpP^S301A^* from a tetracycline-inducible promoter on a multicopy plasmid (34, 39). We observed that increased expression of *pbpP^+^* or *pbpP^S301A^* leads to a dose-dependent increase in the expression of P*_sigP_-lacZ*, in the absence of β-lactams (Fig. 3A). We also found that the addition of cefoxitin led to a further increase in P*_sigP_-lacZ* expression when *pbpP^+^* was overexpressed (Fig. S4B). We noted increased basal levels of *P_sigP_-lacZ* expression in the absence of anhydrotetracycline (ATc) and concluded that this is likely due to leaky expression of *P_tet_-pbpP* and *P_tet_-pbpP^S301A^* (Fig. 3A and Fig. S4B). These data suggest that the overexpression of both the WT and the active-site mutant (S301A) can activate σ^P^ even in the absence of β-lactams. We interpret this to mean that the requirement for β-lactam binding to PbpP can be compensated for by increased levels of PbpP; however, β-lactam binding to PbpP further enhances σ^P^ activation (Fig. S4B). The activation of σ^P^ in WT cells is likely not due to β-lactam-induced *pbpP* transcription as the expression of *pbpP* is not induced by β-lactams (Fig. S1B and C). The *pbpP^S301A^* mutant also fails to induce σ^P^ activation when expressed under the control of its native promoter, further suggesting that *pbpP* is not induced by β-lactams (Fig. 2B).

10.1128/mBio.00179-21.4 FIG S4 σ^P^ activation by overexpression of PbpP is enhanced by cefoxitin and causes degradation of RsiP. (A) Overexpression of *pbpP* causes degradation of RsiP (color version of Fig. 3B). All strains harbor IPTG-inducible *gfp*-*rsiP* (*P_IPTG_-gfp-rsiP*) integrated at the ICE*Bs1* site (38) plus either the empty vector (EV) (EBT744), *P_tet_-pbpP^+^* (EBT742), or *P_tet_-pbpP^S301A^* (EBT1144). Strains were grown to mid-log phase with 1 mM IPTG and increasing concentrations of ATc. The cells were concentrated, resuspended in sample buffer, and separated by SDS-PAGE. The immunoblot was probed with antisera against GFP. Streptavidin IR680LT was used to detect HD73_4231 (PycA homolog), which served as a loading control (51, 52). A color blot showing both anti-GFP and streptavidin on a single gel is shown. (B) σ^P^ activation by overexpression of PbpP is enhanced by cefoxitin. All strains contain the reporter *P_sigP_-lacZ* and the relevant genotypes WT EV (pAH9) (EBT169), Δ*pbpP*/EV (EBT344), and Δ*pbpP*/*pbpP^+^* (EBT327) and contain *P_sigP_-lacZ* inserted in *thrC* to report the activity of σ^P^. The cultures were grown to mid-log phase (1.2 to 1.5), washed, and resuspended with anhydrotetracycline (ATc) with or without cefoxitin (Cef). The cultures were incubated for 1 h, and β-galactosidase activity was measured. Download FIG S4, PDF file, 0.08 MB. Copyright © 2021 Nauta et al. 2021 Nauta et al. https://creativecommons.org/licenses/by/4.0/ This content is distributed under the terms of the Creative Commons Attribution 4.0 International license.

Since the loss of PbpP results in little to no degradation of RsiP in the presence of β-lactams, we tested if the increased expression of *pbpP* leads to the degradation of RsiP in the absence of β-lactams. We introduced *P_tet_*-*pbpP^+^* or *P_tet_*-*pbpP^S301A^* into a strain containing IPTG-inducible *gfp*-*rsiP*. We found that the overexpression of PbpP and PbpP^S301A^ leads to decreases in full-length GFP-RsiP levels, suggesting that PbpP can induce RsiP degradation and, thus, σ^P^ activation (Fig. 3B). This suggests that PbpP controls σ^P^ activation by controlling RsiP degradation.

### PbpP is likely not the site 1 protease for RsiP.

**FIG 4:**
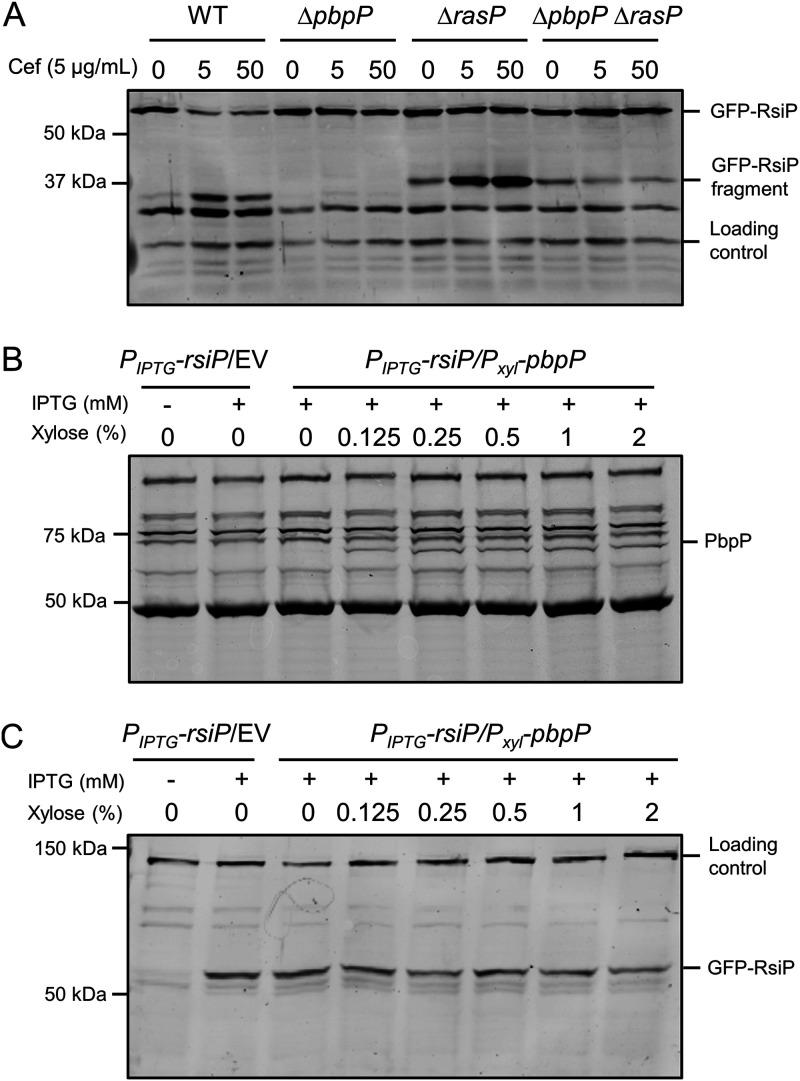
PbpP is not the site 1 protease. (A) Basal levels of site 1 cleavage of RsiP occur in the absence of PbpP. All strains contain *P_IPTG_-gfp-rsiP* and the following relevant genotypes: WT (EBT936), Δ*rasP* (EBT939), Δ*pbpP* (EBT937), and Δ*pbpP* Δ*rasP* (EBT1120). The strains were grown to mid-log phase with IPTG and incubated without or with cefoxitin (5 or 50 μg/ml). The samples were analyzed by immunoblotting using anti-GFP antisera. Streptavidin IR680LT was used to detect AccB (HD73_4487), which served as a loading control (51, 52). A color blot showing both anti-GFP and streptavidin on a single gel is shown in Fig. S5A in the supplemental material. (B) PbpP is produced in B. subtilis. All strains contained *amyE*::*P_IPTG_-gfp-rsiP* with the relevant genotypes WT (CDE3147) and *thrC*::*P_xyl_-pbpP* (EBT756) and were grown to mid-log phase with IPTG at 0.01 mM and increasing concentrations of xylose (0.125 to 2% xylose). At mid-log phase, 1-ml aliquots were concentrated, washed, and resuspended in Bocillin-FL (50 μg/ml) for 30 min at RT. A color blot showing both Bocillin-FL and the ladder on a single gel is shown in Fig. S5B. (C) Samples from panel B were probed with anti-GFP antisera to detect GFP-RsiP, and streptavidin IR680LT was used to detect the PycA homolog, which served as a loading control (51, 52). A color blot showing both anti-GFP and streptavidin on a single gel is shown in Fig. S5C.

The site 1 protease required for initiating RsiP degradation has not yet been identified. Since PbpP is required for site 1 cleavage of RsiP, the possibility exists that PbpP is the site 1 protease. We sought to determine if basal-level site 1 cleavage occurred in the absence of *pbpP*, which would suggest that another protein can cleave RsiP. Since site 2 cleavage is rapid (34), we expressed *gfp-rsiP* in a Δ*pbpP* Δ*rasP* double mutant, which should allow the buildup of any GFP-RsiP site 1 cleavage product. We observed the accumulation of a band corresponding to a GFP-RsiP fragment in the Δ*rasP* mutant in the absence of cefoxitin, and the intensity of this band increased in the presence of cefoxitin (Fig. 4A). We observed the same band in the Δ*pbpP* Δ*rasP* mutant; however, the band did not increase in the presence of cefoxitin. We concluded that in a Δ*pbpP* Δ*rasP* mutant, there is a basal level of site 1 cleavage of RsiP occurring in the presence and absence of cefoxitin (Fig. 4A). This suggests that site 1 cleavage can occur in the absence of PbpP, but it is not β-lactam inducible. Presumably, in this strain, the unidentified site 1 protease still retains its basal level of activity but cannot be further activated in the presence of cefoxitin due to the absence of PbpP.

10.1128/mBio.00179-21.5 FIG S5 PbpP is not the site 1 protease (color version of Fig. 4). (A) Basal levels of site 1 cleavage of RsiP occur in the absence of PbpP. All strains contain *P_IPTG_-gfp-rsiP* and the following relevant genotypes: WT (EBT936), Δ*rasP* (EBT939), Δ*pbpP* (EBT937), and Δ*pbpP* Δ*rasP* (EBT1120). The strains were grown to mid-log phase with IPTG and incubated without or with cefoxitin (5 or 50 μg/ml). The samples were analyzed by immunoblotting using antisera against GFP. Streptavidin IR680LT was used to detect AccB (HD73_4487), which served as a loading control (51, 52). A color blot showing both anti-GFP and streptavidin on a single gel is shown. (B) PbpP is produced in B. subtilis. All strains containing *amyE*::*P_IPTG_-gfp-rsiP* with the relevant genotype, WT (CDE3147) and *thrC*::*P_xyl_-pbpP* (EBT756), were grown to mid-log phase with IPTG at 0.01 mM and increasing concentrations of xylose (0.125 to 2% xylose). At mid-log phase, 1-ml aliquots were concentrated, washed, and resuspended in Bocillin-FL (50 μg/ml) for 30 min at RT. A color blot showing both Bocillin-FL and the ladder on a single gel is shown. (C) Samples from panel B were probed with anti-GFP antisera to detect GFP-RsiP, and streptavidin IR680LT was used to detect the PycA homolog, which served as a loading control (51, 52). A color blot showing both anti-GFP and streptavidin on a single gel is shown. Download FIG S5, PDF file, 0.2 MB. Copyright © 2021 Nauta et al. 2021 Nauta et al. https://creativecommons.org/licenses/by/4.0/ This content is distributed under the terms of the Creative Commons Attribution 4.0 International license.

To test if PbpP is sufficient for site 1 cleavage of RsiP, we introduced IPTG-inducible *gfp*-*rsiP* into the Bacillus subtilis chromosome (which does not encode a homolog of *sigP* or *rsiP*) and expressed *pbpP* using a xylose-inducible promoter. We grew the cells in the presence of 0.01 mM IPTG and increasing concentrations of xylose. We asked if PbpP was expressed and presumably properly folded by labeling with the fluorescent β-lactam Bocillin-FL. We observed a fluorescent band corresponding to PbpP that increased in intensity with increasing concentrations of xylose (Fig. 4B). We also monitored GFP-RsiP levels by performing immunoblot analysis using anti-GFP antisera. We did not observe degradation or a decrease in RsiP levels even at the highest levels of PbpP, indicating that PbpP is not sufficient for RsiP degradation in B. subtilis (Fig. 4C). Taken together, these data lead us to conclude that PbpP is not the site 1 protease but is required for sensing of β-lactams in B. thuringiensis.

### Affinities of β-lactams for PbpP do not correlate with their ability to activate σ^P^.

**FIG 5:**
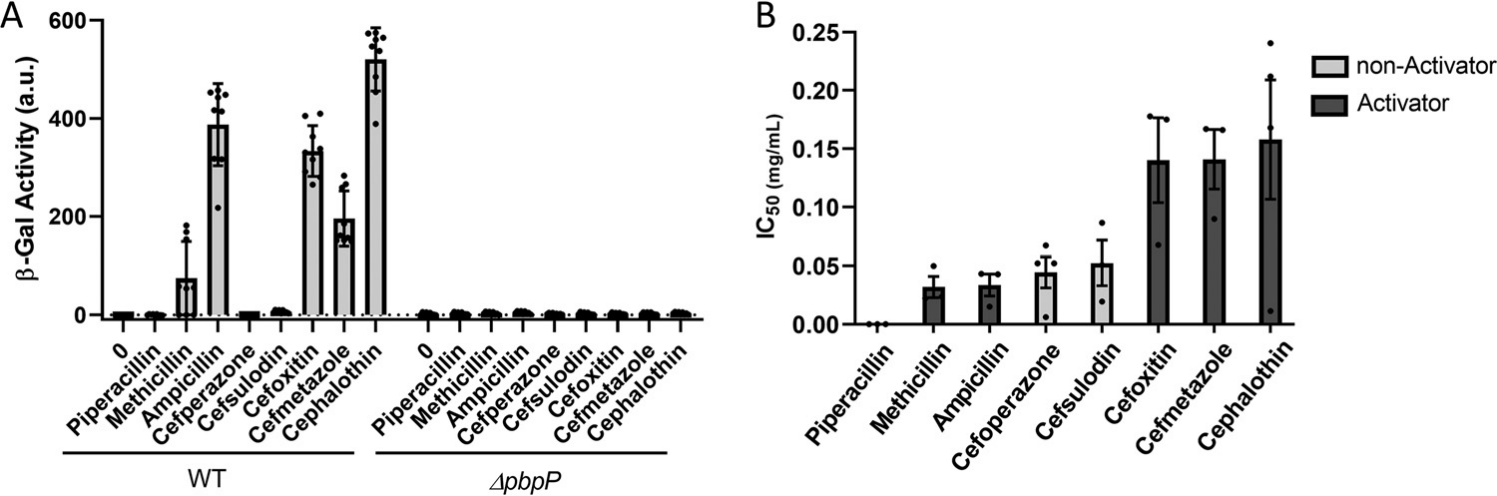
β-Lactams bind PbpP with similar affinities. (A) A subset of β-lactams activate σ^P^ and require PbpP for σ^P^ activation. Both WT (THE2549) and Δ*pbpP* (EBT151) strains contained *P_sigP_-lacZ*. Mid-log-phase cells were resuspended in 1 ml of LB medium with 2 μg/ml of the β-lactam indicated and incubated with aeration for 1 h, and β-galactosidase activity was determined. (B) The activating β-lactams do not have a higher affinity for PbpP than nonactivating β-lactams. The Δ*sigP-rsiP*/*P_tet_-pbpP* strain (EBT509) was subcultured 1:50 and grown to mid-log phase with ATc (100 ng/ml) at 37°C. The cells were washed in PBS and resuspended in 10-fold dilutions of β-lactams in PBS. The cells were incubated for 30 min at ∼22°C, pelleted, and resuspended in Bocillin-FL (50 μg/ml) for 15 min at ∼22°C. The cells were pelleted, resuspended in sample buffer, and separated by SDS-PAGE. Bocillin-FL-bound proteins were detected by excitation at 488 nm and detection at 518 nm. The band intensities corresponding to PbpP were measured three times for each gel and then averaged. The data shown are the averages from three independent gels for each antibiotic. GraphPad Prism 8.1.2 was used to calculate the IC_50_s for each antibiotic using a log (inhibitor)-versus-response-variable slope (four parameters) and least-square (ordinary) fit. The individual Bocillin-FL inhibition curves for each β-lactam are shown in Fig. S6 in the supplemental material, and an example of each gel showing decreasing PBP band fluorescence intensities with increasing concentrations of β-lactams is shown in Fig. S7.

Since PbpP is likely not acting as the site 1 protease, we hypothesized that PbpP functions as a sensor that binds β-lactams and subsequentially activates σ^P^. Therefore, we hypothesized that the reason why some β-lactams do not activate σ^P^ is that they have a lower affinity for PbpP. To test this hypothesis, we determined the affinity of PbpP for eight different β-lactams by modifying a Bocillin-FL inhibition experiment previously described by Kocaoglu and colleagues (40). We calculated the 50% inhibitory concentration (IC_50_) (the concentration of β-lactam at which 50% of Bocillin-FL labeling of PbpP is inhibited) to determine the binding affinity of different β-lactams. We found that while the β-lactams had different IC_50_s for PbpP, the differences did not correlate with the ability of the β-lactams to activate σ^P^ (Fig. 5A and B). For example, we found that some of the nonactivating β-lactams (cefoperazone and cefsulodin) had IC_50_s similar to those of activating β-lactams (Fig. 5A and B). Thus, the disparity in the β-lactams’ ability to activate σ^P^ is not simply due to the inability of PbpP to bind different β-lactams. These data also suggest that simple binding of any β-lactam to PbpP is not sufficient for σ^P^ activation.

10.1128/mBio.00179-21.6 FIG S6 β-Lactam curves for calculating IC_50_s. The Δ*sigP-rsiP*/*P_tet_-pbpP* strain (EBT509) was subcultured at 1:50 and grown to mid-log phase with ATc at 100 ng/ml at 37°C. The cells were washed in PBS and resuspended in 10-fold dilutions of β-lactams in PBS. The cells were incubated for 30 min at RT, pelleted, and resuspended in Bocillin-FL at 50 μg/ml for 15 min at RT. The cells were pelleted and resuspended in sample buffer. The samples were sonicated, boiled, and run on a 12% SDS-PAGE gel. The gel was imaged by excitation at 488 nm and detection at 518 nm as described in Materials and Methods. The band intensities corresponding to PbpP were measured three times and then averaged. The experiment was repeated in triplicate for each antibiotic, and the data shown are the averages from the three replicates. GraphPad Prism 8.1.2 was used to calculate the IC_50_s for each antibiotic using a log (inhibitor)-versus-response-variable slope (four parameters) and least-square (ordinary) fit. Download FIG S6, PDF file, 0.2 MB. Copyright © 2021 Nauta et al. 2021 Nauta et al. https://creativecommons.org/licenses/by/4.0/ This content is distributed under the terms of the Creative Commons Attribution 4.0 International license.

10.1128/mBio.00179-21.7 FIG S7 Representative SDS-PAGE gels corresponding to Fig. 5. The β-lactams that activate σ^P^ do not have a higher affinity for PbpP than the β-lactams that do not activate it. The Δ*sigP-rsiP*/*P_tet_-pbpP* strain (EBT509) was subcultured at 1:50 and grown to mid-log phase with ATc at 100 ng/ml at 37°C. The cells were washed in PBS and resuspended in 10-fold dilutions of β-lactams in PBS. The cells were incubated for 30 min at RT, pelleted, and resuspended in Bocillin-FL at 50 μg/ml for 15 min at RT. The cells were pelleted and resuspended in sample buffer. The samples were sonicated, boiled, and run on a 12% SDS-PAGE gel. The gel was imaged by excitation at 488 nm and detection at 518 nm as described in Materials and Methods. One SDS-PAGE gel representative of each antibiotic is shown here. Download FIG S7, PDF file, 1.2 MB. Copyright © 2021 Nauta et al. 2021 Nauta et al. https://creativecommons.org/licenses/by/4.0/ This content is distributed under the terms of the Creative Commons Attribution 4.0 International license.

### Cefsulodin inhibits activation of σ^P^ by cefoxitin.

**FIG 6:**
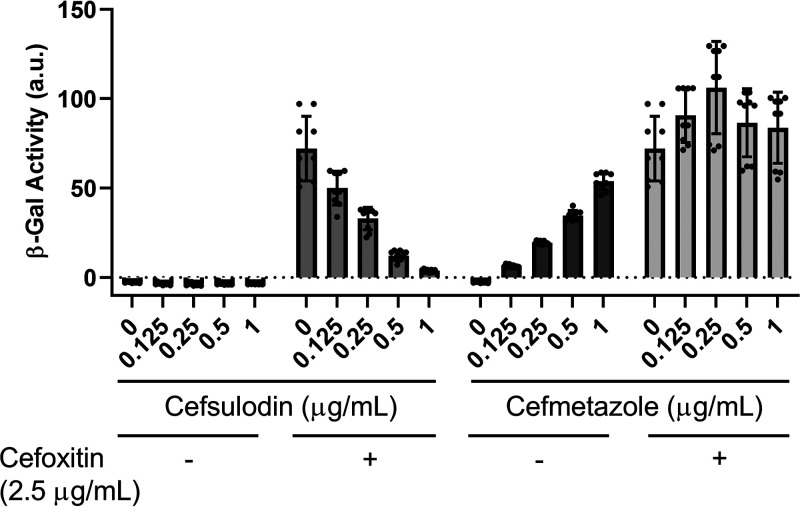
Cefsulodin inhibits activation of σ^P^ by inhibiting the active site of PbpP. The WT carrying *P_sigP_-lacZ* (THE2549) was grown to mid-log phase (OD_600_ of 1.2 to 1.4) at 30°C and washed. The cells were resuspended in LB medium with cefsulodin or LB medium with cefmetazole for 5 min. Cefoxitin (2.5 μg/ml) was then added, and the cells were incubated with aeration for 1 h at 37°C. β-Galactosidase activity was measured. Experiments were performed in triplicate, and standard deviations are represented by error bars.

**FIG 7:**
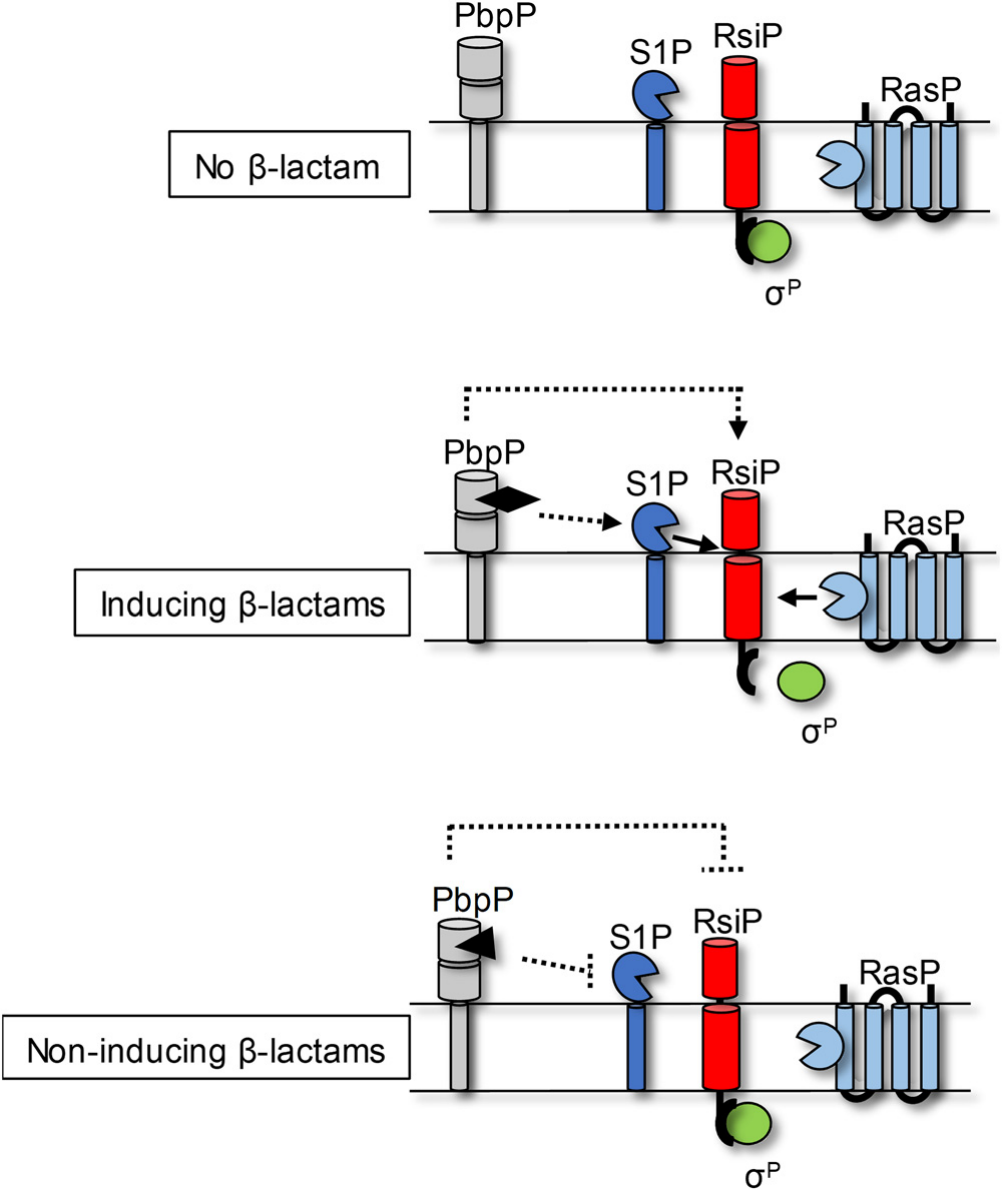
Model of σ^P^ activation incorporating the role of PbpP. The anti-σ factor RsiP (red) sequesters σ^P^ (green) in the absence of β-lactams. In the presence of inducing β-lactams, PbpP (gray) binds the β-lactams, and this interaction results in the activation of the site 1 protease (S1P) (dark blue). After site 1 cleavage of RsiP, RasP (light blue) cleaves RsiP at site 2. This results in the release of σ^P^ from RsiP. In the presence of noninducing β-lactams, the β-lactams bind PbpP; however, this interaction does not induce the activation of the site 1 protease. Furthermore, this interaction inhibits the activation of the site 1 protease by other β-lactams. Dashed lines indicate a possible indirect or direct interaction.

We found that β-lactam binding to PbpP is not sufficient for σ^P^ activation because nonactivating β-lactams covalently bind PbpP with affinities similar to those of the activating β-lactams (i.e., cefsulodin and ampicillin have nearly identical binding affinities for PbpP). We hypothesize that the β-lactams that activate σ^P^ induce a conformational change in PbpP that permits a protein-protein interaction. If this hypothesis were true, the β-lactams that do not activate σ^P^ would be able to inhibit the activation of σ^P^ by occupying the PbpP active site. To test this, we pretreated cells with cefsulodin (a nonactivator of σ^P^) and then added cefoxitin (an activator of σ^P^). We found that cefsulodin inhibited the activation of σ^P^ by cefoxitin in a dose-dependent manner (Fig. 6). We also show that pretreatment with cefmetazole (an activator of σ^P^) does not inhibit activation (Fig. 6). Therefore, nonactivating β-lactams inhibit σ^P^ activation presumably by occupying the active site of PbpP and preventing activating β-lactams from binding PbpP and activating σ^P^ (Fig. 7).

## DISCUSSION

Our data argue that PbpP is a sensor for β-lactams that is required for σ^P^ activation by indirectly promoting the degradation of RsiP (Fig. 7). This is supported by our observation that the loss of the penicillin-binding protein PbpP blocks σ^P^ activation and RsiP degradation. Our data indicate that the binding of a subset of β-lactams to PbpP results in σ^P^ activation. However, inhibition of PbpP transpeptidase activity by β-lactams is not the signal that activates σ^P^ since the transpeptidase mutant PbpP^S301A^ does not activate σ^P^. Interestingly, the overexpression of PbpP and PbpP^S301A^ activates σ^P^ even in the absence of β-lactams; however, PbpP is not the site 1 protease. Together, these results argue that PbpP is a sensor of β-lactams and controls σ^P^ activation.

### PbpP is required for σ^P^ activation.

The principal finding of this work is the demonstration that PbpP is required for the activation of σ^P^ in response to some β-lactams. Based on our findings, we propose the following working model for how PbpP functions as a sensor for β-lactams. In WT cells in the absence of stress, RsiP binds σ^P^ and inhibits σ^P^ activation (Fig. 7). When activating β-lactams are present, they bind the active-site serine of PbpP. The binding of the activating β-lactams results in a conformational change in PbpP that allows it to interact with a component of the σ^P^ system. This interaction initiates regulated intramembrane proteolysis of RsiP and, thus, σ^P^ activation (Fig. 7). This model is supported by ample evidence: (i) deletion of *pbpP* blocks RsiP degradation and σ^P^ activation, (ii) mutants of PbpP unable to bind β-lactams fail to activate σ^P^ in response to β-lactams, and (iii) overexpression of PbpP or PbpP^S301A^ leads to constitutive RsiP degradation and σ^P^ activation. Thus, PbpP plays an essential role in sensing the presence of inducing β-lactams and controlling σ^P^ activation.

### PbpP is not the site 1 protease.

It is possible that PbpP is a site 1 protease that initiates RsiP degradation; however, we think that it is unlikely. While PbpP is required for site 1 cleavage of RsiP in response to β-lactams, the totality of our data does not support PbpP as the site 1 protease. First, the overexpression of PbpP in B. subtilis does not induce the degradation of RsiP as it does in B. thuringiensis. Our data indicate that PbpP is functional, folded, and localized properly when expressed in B. subtilis since it can be labeled on whole cells by Bocillin-FL. This argues that PbpP is not sufficient for site 1 cleavage of RsiP and suggests that an unidentified B. thuringiensis protease is required. Second, in B. thuringiensis, we observed low-level site 1 cleavage of RsiP in the absence of PbpP. This argues that PbpP is not absolutely required for site 1 cleavage. If PbpP were a site 1 protease, there must be a second protease in B. thuringiensis that has low basal activity and cleaves RsiP at site 1 in the absence of PbpP. Finally, PbpP lacks any predicted protease domains. Future work will be required to identify the protease(s) required for site 1 cleavage of RsiP and, thus, σ^P^ activation.

### PbpP is the β-lactam sensor for the σ^P^ system.

We hypothesize that PbpP is the sensor of β-lactams for the σ^P^ system. In support of this, we found that σ^P^ is not activated in the Δ*pbpP* mutant or when *pbpP^S301A^* is expressed from the native *P_pbpP_* promoter. However, the overproduction of either PbpP or PbpP^S301A^ results in the activation of σ^P^ in the absence of β-lactams. This suggests that the overproduction of PbpP can compensate for β-lactam binding to PbpP to activate σ^P^. Importantly, activation of σ^P^ is not due to inhibition of PbpP transpeptidase activity by β-lactams because PbpP^S301A^ is catalytically inactive yet does not result in σ^P^ activation. This loss of σ^P^ activity is not due to an instability of PbpP^S301A^ as it is produced at levels similar to those of WT PbpP. Activation of σ^P^ by β-lactams is not simply due to increased expression of *pbpP* since β-lactams do not induce *pbpP* expression. In addition, if increased expression of *pbpP* in response to β-lactams was responsible for σ^P^ activation, then we would have expected the *pbpP^S301A^* allele to induce σ^P^ activation when expressed under the control of the native P*_pbpP_* promoter. Taken together, these data suggest that PbpP interacts with some component of the signal transduction system.

In support of this hypothesis, we found that a subset of activating β-lactams bind PbpP with affinities similar to those of nonactivating β-lactams. We found that cefsulodin, a nonactivating β-lactam, can inhibit the activation of σ^P^ by an activating β-lactam, cefoxitin, presumably by competing for the active-site serine of PbpP. We hypothesize that nonactivating β-lactams do not induce the appropriate conformational change in PbpP to render it active and able to interact with its target. One obvious target for PbpP interaction is the anti-σ itself. However, we did not observe an interaction between the extracellular domains of RsiP^76–275^ and PbpP^35–586^ *in vitro* using a copurification assay (see Fig. S8 in the supplemental material). This raises the possibility that PbpP interacts with another protein like the as-yet-unidentified site 1 protease. Alternatively, it may interact indirectly with RsiP or the site 1 protease via an unknown protein. Future work will need to determine what PbpP interactions drive RsiP degradation and, thus, σ^P^ activation.

10.1128/mBio.00179-21.8 FIG S8 PbpP does not bind RsiP *in vitro.* 6×His-RsiP was purified from E. coli. PbpP was produced *in vitro* using a PURExpress *in vitro* protein synthesis kit and then run over a nickel column to eliminate 6×His-tagged proteins. An aliquot of *in vitro*-produced PbpP was incubated without RsiP (wells 1 and 2). 6×His-RsiP alone is in wells 3 and 4. 6×His-RsiP incubated with PbpP is in wells 5 to 7. 6×His-RsiP incubated with PbpP and cefoxitin (50 mg/ml) is in wells 8 to 10. After incubation, the columns were washed and eluted. The unbound fraction is annotated as the flowthrough (FT), and elution fractions are designated E1 or E2. Download FIG S8, PDF file, 0.2 MB. Copyright © 2021 Nauta et al. 2021 Nauta et al. https://creativecommons.org/licenses/by/4.0/ This content is distributed under the terms of the Creative Commons Attribution 4.0 International license.

### Comparison of the BlaRI response to β-lactams to σ^P^ activation.

While the identification of a PBP required for the activation of an ECF σ factor is novel, there is precedence for a PBP transpeptidase-like domain functioning as a sensor of β-lactams. Found in diverse organisms, including Staphylococcus aureus and Bacillus licheniformis, BlaR1 (MecR1) contains an extracellular transpeptidase-like domain that senses β-lactams and a cytoplasmic protease domain. BlaR1 is a β-lactam sensor that directly binds β-lactams in its extracellular transpeptidase-like domain (41). The covalent bond formed with the β-lactam ring causes a conformational change in BlaR1 that activates the cytoplasmic protease domain (42). The protease domain cleaves the repressor of the β-lactamase operon, BlaI, thus activating the transcription of β-lactamase and increasing resistance to β-lactams (42). While the BlaIR system is clearly not synonymous with σ^P^, it is worth noting that there is precedence for PBP domains that function as sensors of β-lactams.

## MATERIALS AND METHODS

### Media and growth conditions.

All B. thuringiensis strains are isogenic derivatives of AW43, a derivative of B. thuringiensis subsp. *kurstaki* strain HD73 (43). All strains and genotypes can be found in Table 2. All B. thuringiensis strains were grown in or on LB media at 30°C unless otherwise specified. Liquid cultures of B. thuringiensis were grown with agitation in a roller drum. B. thuringiensis strains containing episomal plasmids were grown in LB medium containing chloramphenicol (Cam) (10 μg/ml; Ameresco) or erythromycin (Erm) plus lincomycin (Linc) (MLS) (1 μg/ml Erm [Ameresco] and 25 μg/ml Linc [Research Products International]). E. coli strains were grown at 37°C using LB-ampicillin (Amp) (100 μg/ml; Ameresco) or LB-Cam (10 μg/ml) medium. B. subtilis strains were grown on LB medium with antibiotics (Cam at 10 μg/ml, spectinomycin [Spec] at 100 μg/ml [Amresco], or Erm at 10 μg/ml). To screen for threonine auxotrophy, B. thuringiensis strains were patched onto minimal medium plates without or with threonine (50 μg/ml). The β-galactosidase chromogenic indicator 5-bromo-4-chloro-3-indolyl-β-d-galactopyranoside (X-Gal; Research Products International) was used at a concentration of 100 μg/ml. Anhydrotetracycline (ATc; Sigma) was used at a concentration of 100 ng/ml unless otherwise indicated. IPTG (Research Products International) and xylose (Acros) were used at the concentrations indicated in the figure legends. Additional β-lactams used in β-lactam-binding experiments were used at the concentrations indicated in the figure legends and were acquired from the following sources: cefsulodin, piperacillin, cefmetazole, and cefoxitin from Sigma-Aldrich; cephalothin from Chem-impex International Inc.; methicillin from Alfa Aesar; and cefoperazone from Toronto Research Chemical Inc.

**TABLE 2:** Strains

Strain	Description	Reference or source
B. thuringiensis		
AW43	B. thuringiensis subsp. *kurstaki* HD73 cured of both pAW63 and pHT73; Nal^r^	43
THE2549	AW43 *thrC*::*P_sigP_-lacZ*	34
EBT232	AW43 *thrC*::*P_sigP_-lacZ* Δ*sigP*-*rsiP*	34
EBT151	AW43 *thrC*::*P_sigP_-lacZ* Δ*pbpP*	This study
EBT360	AW43 *thrC*::*P_sigP_*-*lacZ*/pEBT13 (*P_tet_*-*gfp-rsiP*)	34
EBT512	AW43 *thrC*::*P_sigP_*-*lacZ* Δ*pbpP*/pEBT13 (P*_tet_*-*gfp-rsiP*)	This study
EBT366	AW43 *thrC*::*P_sigP_*-*lacZ* Δ*rasP*/pEBT13 (P*_tet_*-*gfp-rsiP*)	34
EBT772	AW43 *thrC*::*P_sigP_-lacZ* Δ*pbpP* ICE*Bs1*::*P_pbpP_-pbpP^S301A^ tetM cat*	This study
EBT773	AW43 *thrC*::*P_sigP_-lacZ* Δ*pbpP* ICE*Bs1*::*P_pbpP_-pbpP^+^ tetM cat*	This study
CDE3214	AW43 *thrC*::*P_sigP_-lacZ* Δ*sigP-rsiP* ICE*Bs1*::*P_IPTG_ tetM cat*	This study
CDE3248	AW43 *thrC*::*P_sigP_-lacZ* Δ*sigP-rsiP* ICE*Bs1*::*P_IPTG_-pbpP^+^ tetM cat*	This study
CDE3243	AW43 *thrC*::*P_sigP_-lacZ* Δ*sigP-rsiP* ICE*Bs1*::*P_IPTG_-pbpP^S301A^ tetM cat*	This study
EBT344	AW43 *thrC*::*P_sigP_-lacZ* Δ*pbpP*/pAH9	This study
EBT327	AW43 *thrC*::*P_sigP_-lacZ* Δ*pbpP*/pEBT20 (*P_tet_-pbpP^+^*)	This study
EBT1145	AW43 *thrC*::*P_sigP_-lacZ* Δ*pbpP*/pCE693 (*P_tet_-pbpP^S301A^*)	This study
EBT744	AW43 *thrC*::*P_sigP_-lacZ* ICE*Bs1*::*P_IPTG_-gfp-rsiP tetM cat*/pAH9	This study
EBT742	AW43 *thrC*::*P_sigP_-lacZ* ICE*Bs1*::*P_IPTG_-gfp-rsiP tetM cat*/pEBT20 (*P_tet_-pbpP^+^*)	This study
EBT1144	AW43 *thrC*::*P_sigP_-lacZ* ICE*Bs1*::*P_IPTG_-gfp-rsiP tetM cat*/pCE693 (*P_tet_-pbpP^S301A^*)	This study
EBT936	AW43 *thrC*::*P_sigP_-lacZ* ICE*Bs1*::*P_IPTG_-gfp-rsiP tetM cat*	This study
EBT937	AW43 *thrC*::*P_sigP_-lacZ* Δ*pbpP* ICE*Bs1*::*P_IPTG_-gfp-rsiP tetM cat*	This study
EBT939	AW43 *thrC*::*P_sigP_-lacZ* Δ*rasP* ICE*Bs1*::*P_IPTG_-gfp-rsiP tetM cat*	This study
EBT1120	AW43 *thrC*::*P_sigP_-lacZ* Δ*pbpP* Δ*rasP* ICE*Bs1*::*P_IPTG_-gfp-rsiP tetM cat*	This study
EBT169	AW43 *thrC*::*P_sigP_-lacZ*/pAH9	34
EBT251	AW43 *thrC*::*P_sigP_-lacZ* Δ*sigP-rsiP*/pAH9	34
EBT275	AW43 *thrC*::*P_sigP_-lacZ*/pEBT10 (*P_pbpP_-pbpP^+^*)	This study
EBT274	AW43 *thrC*::*P_sigP_-lacZ* Δ*sigP-rsiP*/pEBT10 (*P_pbpP_-pbpP^+^*)	This study
EBT276	AW43 *thrC*::*P_sigP_-lacZ* Δ*pbpP*/pEBT10 (*P_pbpP_-pbpP^+^*)	This study
EBT234	AW43 *thrC*::*P_pbpP_-lacZ*	This study
THE2628	AW43 *thrC*::*P_sigP_-lacZ rsiP^1–80^*	34
EBT509	AW43 *thrC*::*P_sigP_-lacZ* Δ*sigP-rsiP*/pEBT20 (*P_tet_-pbpP*^+^)	

B. subtilis		
PY79	Prototrophic derivative of B. subtilis 168	53
CDE3147	PY79 *amyE*::*P_IPTG_-gfp-rsiP*	This study
EBT756	PY79 *amyE*::*P_IPTG_-gfp-rsiP thrC*::*P_xyl_-pbpP*	This study
JAB932	*trpC2 pheA1* Δ(*ydcS-yddM*)::*aphA-3 thrC*::[(*int-yddJ*) Δ*nicK mls*] *alrA*::[(*P_sweet_-rapI*) *spec*]	38
CDE3355	*trpC2 pheA1* Δ(*ydcS-yddM*)::*P_pbpP_-pbpP^S301A^ tetM cat thrC*::[(*int-yddJ*) Δ*nicK mls*] *alrA*::[(*P_sweet_-rapI*) *spec*]	This study
CDE3354	*trpC2 pheA1* Δ(*ydcS-yddM*)::*P_pbpP_-pbpP^+^ tetM cat thrC*::[(*int-yddJ*) Δ*nicK mls*] *alrA*::[(*P_sweet_-rapI*) *spec*]	This study
CDE3226	*trpC2 pheA1* Δ(*ydcS-yddM*)::*P_IPTG_ tetM cat thrC*::[(*int-yddJ*) Δ*nicK mls*] *alrA*::[(*P_sweet_-rapI*) *spec*]	This study
CDE3174	*trpC2 pheA1* Δ(*ydcS-yddM*)::*P_IPTG_-pbpP^+^ tetM cat thrC*::[(*int-yddJ*) Δ*nicK mls*] *alrA*::[(*P_sweet_-rapI*) *spec*]	This study
EBT945	*trpC2 pheA1* Δ(*ydcS-yddM*)::*P_IPTG_-gfp-rsiP tetM cat thrC*::[(*int-yddJ*) Δ*nicK mls*] *alrA*::[(*P_sweet_-rapI*) *spec*]	
CDE3207	*trpC2 pheA1* Δ(*ydcS-yddM*)::*P_IPTG_-pbpP^S301A^ tetM cat thrC*::[(*int-yddJ*) Δ*nicK mls*] *alrA*::[(P*_sweet_-rapI*) *spec*]	This study

E. coli		
OmniMax 2-T1R	F′ [*proAB*^+^ *lacI*^q^ *lacZ*ΔM15 Tn*10*(Tet^r^) Δ(*ccdAB*)] *mcrA* Δ(*mrr-hsdRMS-mcrBC*) ϕ80(*lacZ*)ΔM15 Δ(*lacZYA-argF*)*U169 endA1 recA1 supE44 thi-1 gyrA96 relA1 tonA panD*	Invitrogen
INV110	*endA1 rpsL thr leu thi lacY galK galT ara tomA tsx dam dcm supE44* Δ(*lac-proAB*) [F′ *traD36 proAB lacI*^q^*Z*ΔM15]	Invitrogen
Rosetta DE3	F^−^ *ompT hsdSB*(r_B_^−^ m_B_^−^) *gal dcm* (DE3) pRARE (Cam^r^)	Novagen
CDE2865	pCE593 in Rosetta DE3	This study

### Strain and plasmid construction.

All plasmids are listed in Table 3 and Table S1 in the supplemental material, which includes information relevant to plasmid assembly. Plasmids were constructed by isothermal assembly (44). Regions of plasmids constructed using PCR were verified by DNA sequencing. The oligonucleotide primers used in this work were synthesized by Integrated DNA Technologies (Coralville, IA) and are listed in Table S2. All plasmids were propagated using OmniMax 2-T1R as the cloning host and passaged through the nonmethylating E. coli strain INV110 before being transformed into a B. thuringiensis recipient strain.

**TABLE 3:** Plasmids

Plasmid	Relevant feature(s)	Reference or source
pMAD	ori-pE194ts *amp erm*	45
pAH9	ori-pE194 *P_sarA_-mcherry amp erm*	39
pJAB980	ICE::*P_IPTG_-gfp amp cat*	38
pAC68	*thrC*::*P_xyl_ amp erm*	Arnaud Chastanet
pDR111	*amyE*::*P_IPTG_ amp spec*	David Rudner
pRAN332	*P_tet_-gfp cat*	54
pEBT13	*P_tet_-gfp-rsiP amp erm*	34
pTHE950	pE194ts ‘*thrC lacZ thrB*’ *cat*	34
pTHE955	pE194ts ‘*thrC P_pbpP_-lacZ thrB*’ *cat*	This study
pEBT2	ori-pE194ts Δ*pbpP amp erm*	This study
pEBT10	ori-pE194 *P_pbpP_-pbpP^+^ amp erm*	This study
pEBT20	ori-pE194 *P_tet_-pbpP^+^ amp erm*	This study
pCE693	ori-pE194 *P_tet_-pbpP^S301A^ amp erm*	This study
pCE784	ICE*Bs1*::*P_pbpP_-pbpP^+^ amp cat*	This study
pCE785	ICE*Bs1*::*P_pbpP_-pbpP*^S301A^ *amp cat*	This study
pCE707	ICE*Bs1*::*P_IPTG_-pbpP^+^ amp cat*	This study
pCE726	ICE*Bs1*::*P_IPTG_-pbpP^301A^ amp cat*	This study
pCE755	*thrC*::*P_xyl_-pbpP^+^ amp erm*	This study
pCE695	*amyE*::*P_IPTG_*-*gfp-rsiP amp spec*	This study
pCE698	ICE*Bs1*::*P_IPTG_*-*gfp-rsiP amp cat*	This study
pCE697	ICE*Bs1*::*P_IPTG_ amp cat*	This study
pCE593	P_T7_-6×His-*rsiP^76–275^ amp*	This study
pCE830	P_T7_-*pbpP^35–586^ amp*	This study

10.1128/mBio.00179-21.9 TABLE S1 Plasmids used in this study. Download Table S1, PDF file, 0.2 MB. Copyright © 2021 Nauta et al. 2021 Nauta et al. https://creativecommons.org/licenses/by/4.0/ This content is distributed under the terms of the Creative Commons Attribution 4.0 International license.

10.1128/mBio.00179-21.10 TABLE S2 Primers used in this study. Download Table S2, PDF file, 0.1 MB. Copyright © 2021 Nauta et al. 2021 Nauta et al. https://creativecommons.org/licenses/by/4.0/ This content is distributed under the terms of the Creative Commons Attribution 4.0 International license.

To construct deletion mutants, we cloned 1 kb of DNA upstream and 1 kb downstream of the site of the desired deletion using primers listed in Table S2 into the temperature-sensitive pMAD plasmid (erythromycin resistant) between the BglII and EcoRI sites (45). Mutants were constructed by shifting temperatures as previously described (45).

B. subtilis ICE*Bs1* conjugation strains were constructed by transforming JAB932 as previously described (38). The resulting transformants or donor strains were grown in LB medium with d-alanine (100 μg/ml) for 2 h, at which point 1% xylose was added and cells were grown for 1 h. Recipient strains of B. thuringiensis were grown to an optical density at 600 nm (OD_600_) of ∼0.8. The donor and recipient strains were mixed at equal concentrations, plated on LB medium containing d-alanine (100 μg/ml), and incubated for 6 h. Transconjugants were isolated by plating on LB plates containing chloramphenicol.

### B. thuringiensis DNA transformation.

Plasmids were introduced into B. thuringiensis by electroporation (46, 47). Briefly, recipient cells were grown to late log phase at 37°C from a fresh plate. For each transformation, cells (1.5 ml) were pelleted by centrifugation (8,000 rpm) and washed twice in room-temperature (RT) sterile water. After careful removal of all residual water, 100 μl of filter-sterilized 40% polyethylene glycol 6000 (PEG 6000; Sigma) was used to gently resuspend cells. Approximately 2 to 10 μl of unmethylated DNA (>50 ng/μl) was added to cells and transferred to a 0.4-cm-gap electroporation cuvette (Bio-Rad). Cells were exposed to 2.5 kV for 4 to 6 ms. LB medium was immediately added, and cells were incubated at 30°C for 1 to 2 h prior to plating on selective media.

### β-Galactosidase assays.

To quantify expression from the *sigP* promoter, we measured the β-galactosidase activity of cells containing a *P_sigP_*-*lacZ* promoter fusion. Cultures grown overnight were diluted 1:50 in fresh LB medium and incubated to mid-log phase (OD of 0.8 to 1.5) at 30°C with or without ATc or IPTG. One milliliter of each subculture was pelleted (8,000 rpm), washed (in LB broth), and resuspended in 1 ml LB broth lacking or including specified antibiotics. After 1 h of incubation at 37°C, 1 ml of each sample was pelleted and resuspended in 1 ml of Z-buffer (0.06 M Na_2_HPO_4_, 0.04 M NaH_2_PO_4_*H_2_O, 0.01 M KCl, 0.001 M MgSO_4_). Cells were permeabilized by mixing with 16 μl of chloroform and 16 μl of 2% Sarkosyl (27, 48). Permeabilized cells (50 μl) were mixed with 100 μl of Z-buffer and 50 μl of 2 mg/ml chlorophenol red-β-d-galactopyranoside (CPRG; Research Products International) (50 μl), which is considerably more sensitive than X-Gal (49). The OD_578_ was measured over time using an Infinite M200 Pro plate reader (Tecan). β-Galactosidase activity units [(micromoles of chlorophenol red formed per minute) × 10^3^/(OD_600_ × milliliters of cell suspension)] were calculated as previously described (50). Experiments were performed in technical and biological triplicate, with the means and standard deviations shown.

### MIC assay.

To determine the MICs for various antibiotics, we diluted cultures of bacteria grown overnight (washed in LB medium) 1:1,000 in medium containing 2-fold dilutions of each antibiotic. All MIC experiments were performed in round-bottom 96-well plates. Each experiment was performed in triplicate, and the cultures were allowed to incubate for 24 h at 37°C before observing growth or no growth by centrifuging the plates at 1,000 rpm for 5 minutes and observing the presence or absence of pellets.

### Immunoblot analysis.

Samples were electrophoresed on a 15% SDS-polyacrylamide gel, and proteins were then blotted onto a nitrocellulose membrane (GE Healthcare, Amersham). Nitrocellulose was blocked with 5% bovine serum albumin (BSA), and proteins were detected with a 1:10,000 dilution anti-GFP antisera. Streptavidin IR680LT (1:10,000) was used to detect two biotin-containing proteins, PycA (HD73_4231) and AccB (HD73_4487), which served as loading controls (51, 52). To detect primary antibodies, the blots were incubated with a 1:10,000 dilution of goat anti-rabbit IR800CW (Li-Cor) and imaged on an Odyssey CLx scanner (Li-Cor) or Azure Sapphire (Azure Biosystems). All immunoblots were performed at room temperature a minimum of three times, with a representative example shown.

### Bocillin-FL labeling assay.

Cultures grown overnight at 30°C were diluted 1:50 and grown to an OD of ∼1.0. The cultures were aliquoted in 1-ml aliquots and pelleted at 8,000 rpm. The cells were washed twice in 500 μl of 1× phosphate-buffered saline (PBS) and resuspended in either 50 μl of 50 μg/ml Bocillin-FL (Thermo Fisher) or 50 μl of 10-fold dilutions of β-lactams (0.0005 to 5,000 μg/ml). The samples resuspended in β-lactams were incubated for 30 min at room temperature and then pelleted and resuspended in 50 μl or 50 μg/ml Boc-FL for 15 min. After incubation in Boc-FL, all the samples were pelleted and resuspended in 200 μl sample buffer with 5% β-mercaptoethanol (βME). The samples were sonicated, heated, and electrophoresed on a 12% polyacrylamide gel. The gels were imaged on an Azure Sapphire system (AzureBiosystems) by excitation at 488 nm and detection at 518 nm. The Bocillin-FL labeling experiment was performed in biological triplicate for each antibiotic, and the Bocillin-FL intensity for the PbpP band was quantified on each gel. The average intensity was used to calculate the IC_50_ using GraphPad Prism, with means and standard errors or deviations shown.
