# Supplementary material for: The Penicillin-Binding Protein PbpP Is a Sensor of β-Lactams and Is Required for Activation of the Extracytoplasmic Function σ Factor σP in Bacillus thuringiensis
Source: mBio. 2021 Mar 23;12(2):e00179-21. doi: 10.1128/mBio.00179-21 (PMC8092216; doi:10.1128/mBio.00179-21)
Supplement: FIG S2 [file mBio.00179-21-sf002.pdf]

Figure S2

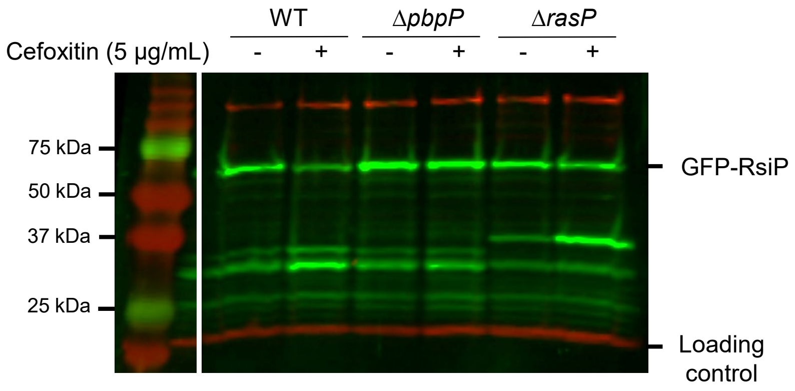

Figure S2: Color version of Figure 1D: PbpP is required for cefoxitin-induced degradation of RsiP. (A) All strains contain the plasmid pBT13 (P_{tet}-*gfp-rsiP*) and the following relevant genotypes: WT (THE360), *ΔpbpP* (EBT512), and *ΔrasP* (EBT366). The strains were grown to mid-log at 37 °C in ATc (100 ng/ml) concentrated and resuspended in 100 µL of LB or LB with cefoxitin (5 µg/ml) for 1 hour. The immunoblot was performed as described in the materials and method using antisera against GFP. Streptavidin IR680LT was used to detect AccB (HD73_4487), which served as a loading control (51, 52). The color blot showing both anti-GFP and streptavidin on a single gel is shown in Fig. S2. Numbers at right indicate molecular masses in kilodaltons of the ladder.
