# Supplementary figures and images for: The Penicillin-Binding Protein PbpP Is a Sensor of β-Lactams and Is Required for Activation of the Extracytoplasmic Function σ Factor σP in Bacillus thuringiensis
Source: mBio. 2021 Mar 23;12(2):e00179-21. doi: 10.1128/mBio.00179-21 (PMC8092216; doi:10.1128/mBio.00179-21)

Figure S1

A

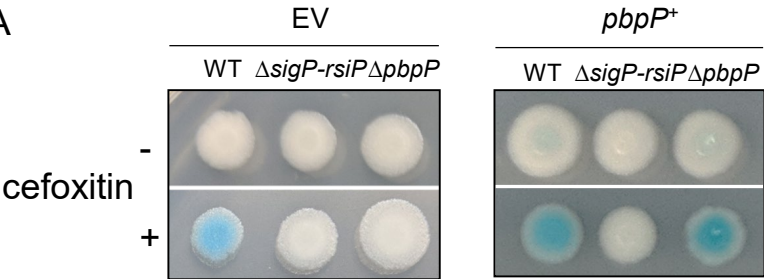

B

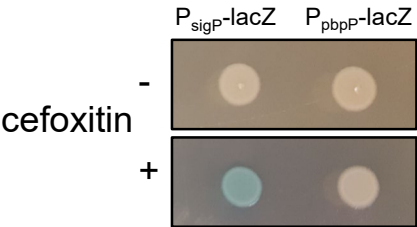

C

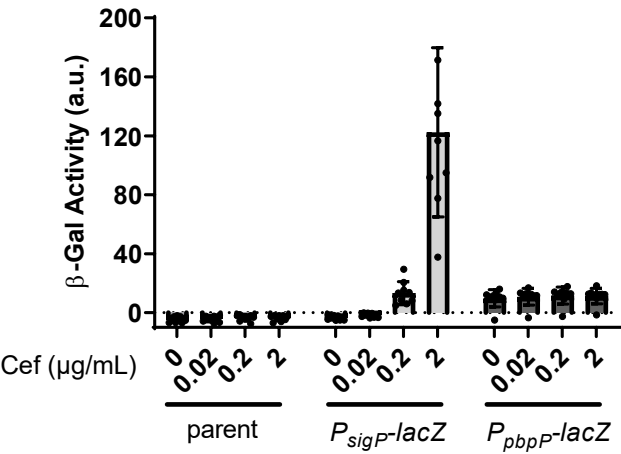

Supplement: FIG S1 [file mBio.00179-21-sf001.pdf]

FIG S4:

A

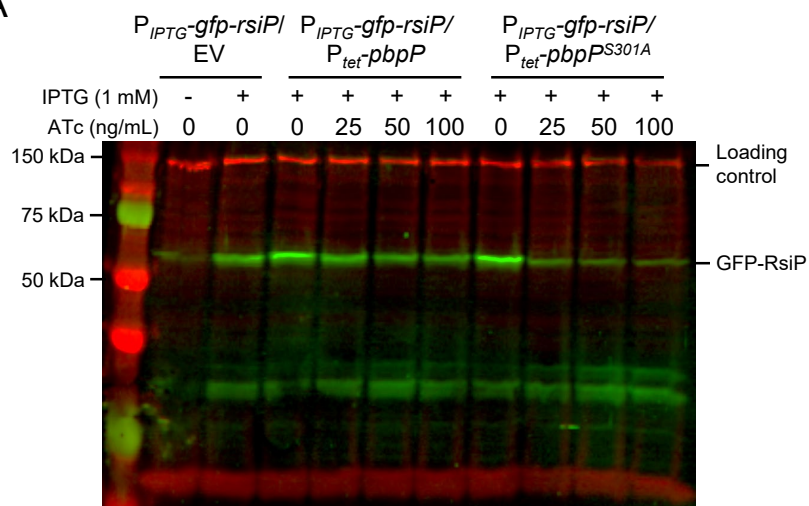

B

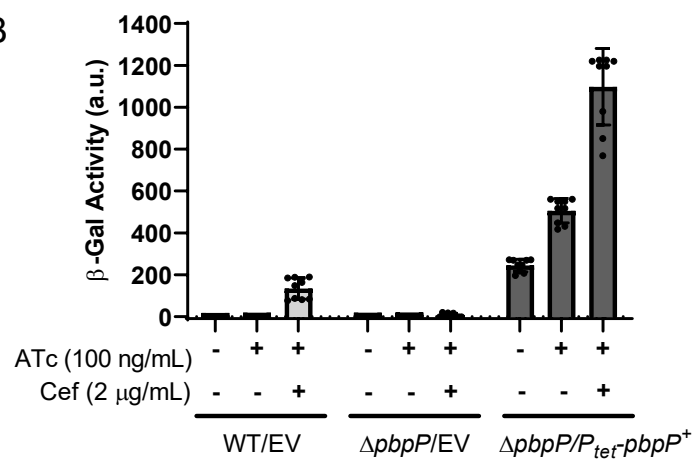

Supplement: FIG S4 [file mBio.00179-21-sf004.pdf]

Figure S5

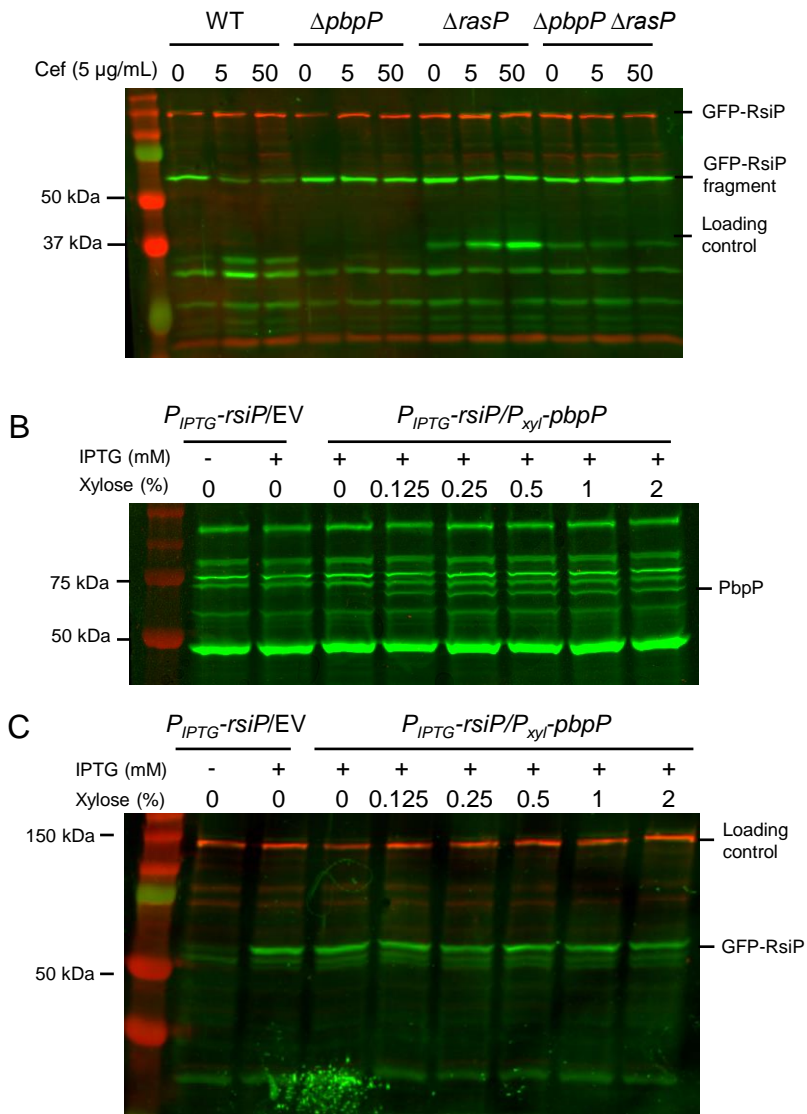

Supplement: FIG S5 [file mBio.00179-21-sf005.pdf]
