# Supplementary material for: The Penicillin-Binding Protein PbpP Is a Sensor of β-Lactams and Is Required for Activation of the Extracytoplasmic Function σ Factor σP in Bacillus thuringiensis
Source: mBio. 2021 Mar 23;12(2):e00179-21. doi: 10.1128/mBio.00179-21 (PMC8092216; doi:10.1128/mBio.00179-21)
Supplement: FIG S3 [file mBio.00179-21-sf003.pdf]

Figure S3

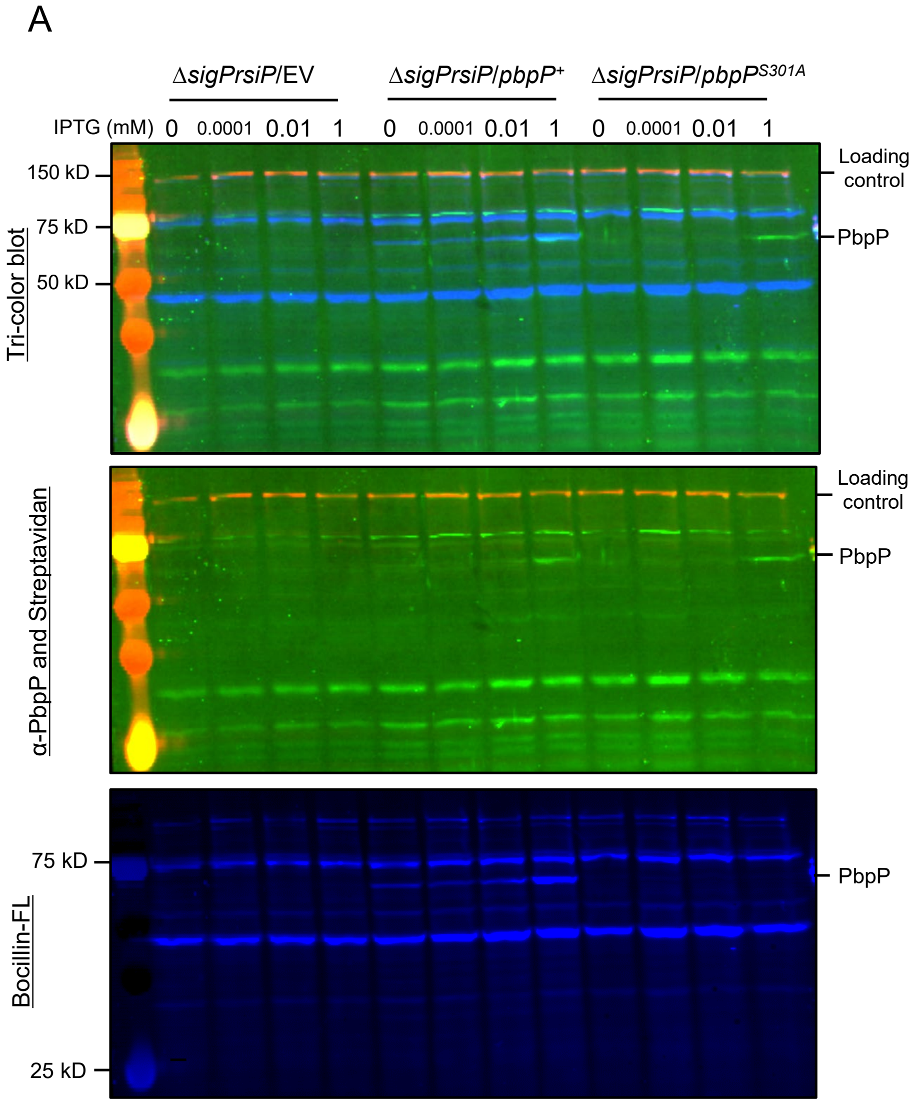

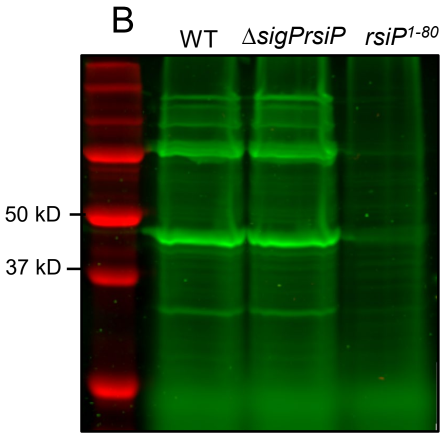

Figure S3. Color version Figure 2A: S301 is the active site serine (A) S301 is the active site serine of PBP*.* All strains contain Δ*sigPrsiP* and then either empty vector (EV) (CDE3214), P_{IPTG}-*pbpP*+ (CDE3248), or P_{IPTG}-*pbpP*^{S301A} (CDE3243). Cells were grown to mid-log with varying concentrations of IPTG. Cells were concentrated, resuspended and incubated with Bocillin-FL (50 µg/ml). The proteins were then separated by SDS-PAGE and the immunoblot was performed as described in the materials and method using antisera against PbpP. Streptavidin IR680LT was used to detect HD73_4231 (PycA homolog), which served as a loading control (51, 52). The color blot showing both anti-PbpP, Bocillin and streptavidin on a single image is top image. A color blot showing anti-PbpP and streptavidin is the middle image The color blot showing Bocillin-FL alone is the bottom image. (B) Activation of σ^P causes degradation of Bocillin-FL. The relevant genotype of strains WT (THE2549), *ΔsigPrsiP* (EBT232), and *rsiP*$^{1-80}$ (THE2628) were grown to mid-log and incubated with Bocillin-FL (50 µg/ml).
