# Supplementary material for: The Penicillin-Binding Protein PbpP Is a Sensor of β-Lactams and Is Required for Activation of the Extracytoplasmic Function σ Factor σP in Bacillus thuringiensis
Source: mBio. 2021 Mar 23;12(2):e00179-21. doi: 10.1128/mBio.00179-21 (PMC8092216; doi:10.1128/mBio.00179-21)
Supplement: FIG S6 [file mBio.00179-21-sf006.pdf]

Figure S6

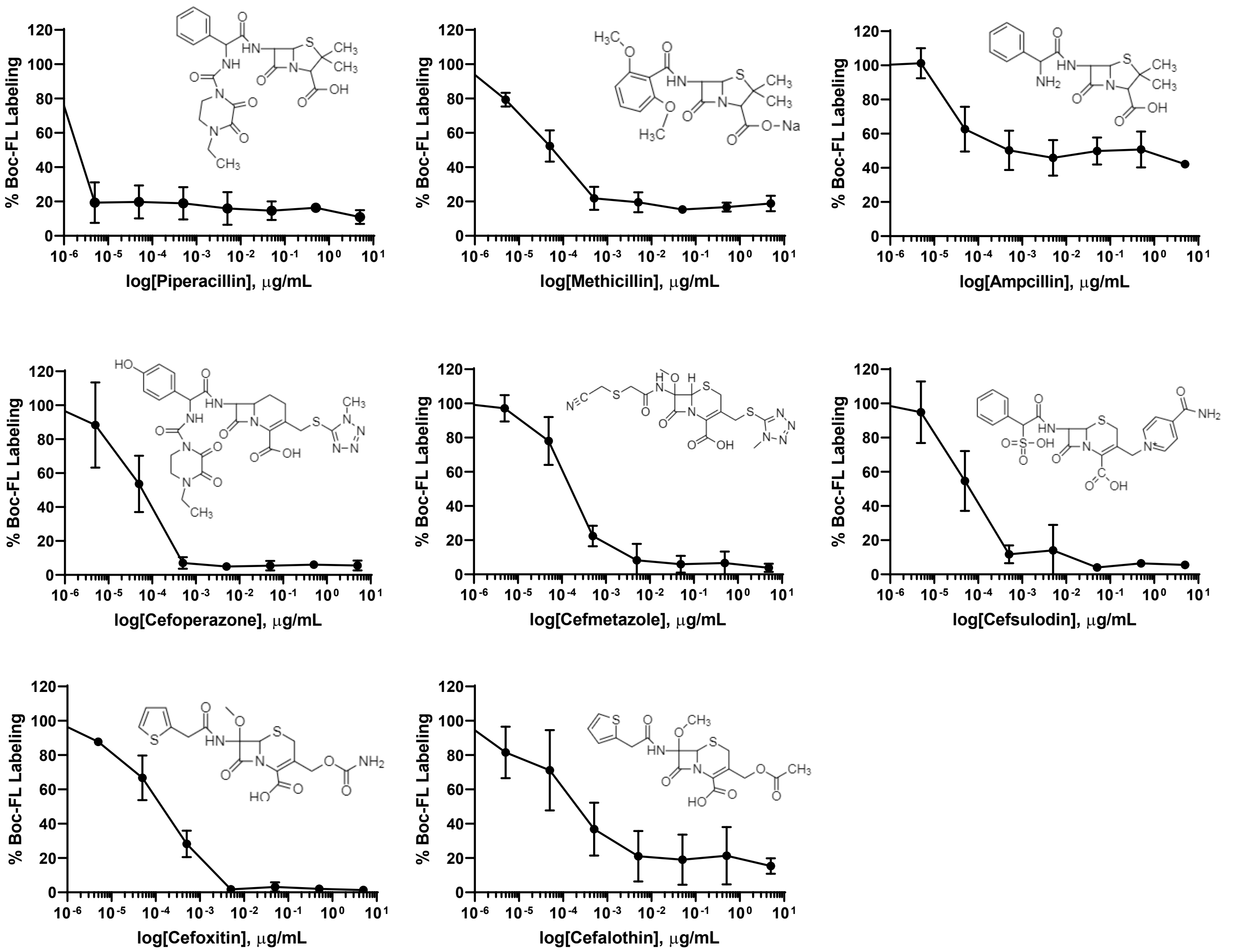

Figure S6. β-lactam curves for calculating IC_{50}s. *ΔsigPrsiP/P_{tet}-pbpP* (EBT509) was subcultured 1:50 and grown to mid-log with ATc 100 ng/mL at 37°C. The cells were washed in PBS and resuspended in 10-fold dilutions of β-lactams in PBS. The cells were incubated for 30 minutes at RT, pelleted, and resuspended in Bocillin-FL 50 μg/mL for 15 minutes at RT. The cells were pelleted and resuspended in sample buffer. The samples were sonicated, boiled, and ran on a 12% SDS PAGE gel. The gel was imaged by exciting at 488 nm and detecting at 518 nm as described in the materials and methods. The band intensities corresponding to PbpP were measured three times and the averaged. The experiment was repeated in triplicate for each antibiotic, the data shown is the average of the three replicates. GraphPad Prism 8.1.2 was used to calculate the IC_{50}s for each antibiotic using a log (inhibitor)-versus-response-variable slope (four parameter) and least square (ordinary) fit.
