# Supplementary material for: The Penicillin-Binding Protein PbpP Is a Sensor of β-Lactams and Is Required for Activation of the Extracytoplasmic Function σ Factor σP in Bacillus thuringiensis
Source: mBio. 2021 Mar 23;12(2):e00179-21. doi: 10.1128/mBio.00179-21 (PMC8092216; doi:10.1128/mBio.00179-21)
Supplement: FIG S7 [file mBio.00179-21-sf007.pdf]

Figure S7

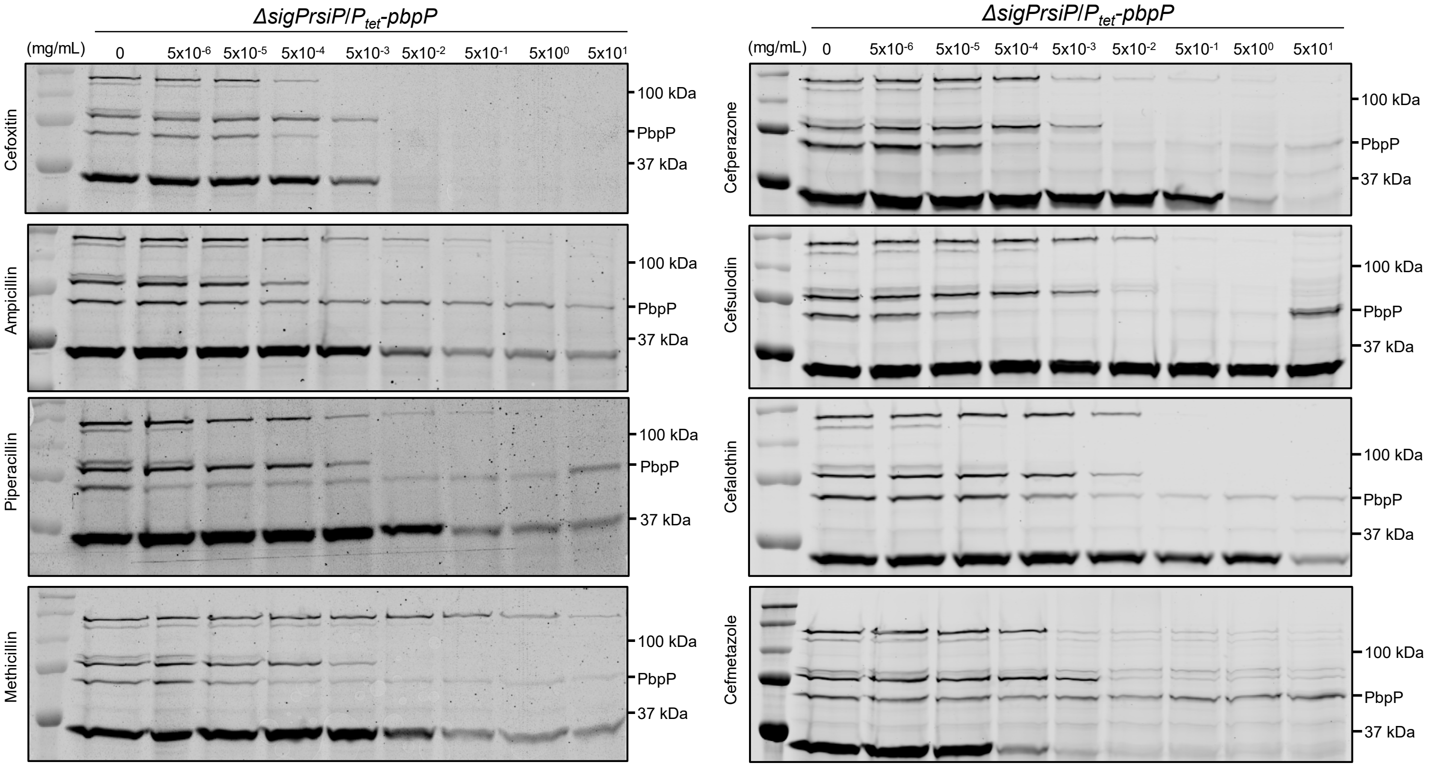

Figure S7. Representative SDS-PAGE gels corresponding to figure 5: The β-lactams that activation σ^{P} do not have a higher affinity for PbpP than the β-lactams that do not activate. *ΔsigPrsiP/P$_{tet}$-pbpP* (EBT509) was subcultured 1:50 and grown to mid-log with ATc 100 ng/mL at 37 °C. The cells were washed in PBS and resuspended in 10-fold dilutions of β-lactams in PBS. The cells were incubated for 30 minutes at RT, pelleted, and resuspended in Bocillin-FL 50 μg/mL for 15 minutes at RT. The cells were pelleted and resuspended in sample buffer. The samples were sonicated, boiled, and ran on a 12% SDS PAGE gel. The gel was imaged by exciting at 488 nm and detecting at 518 nm as described in the materials and methods. One SDS-PAGE gel representative for each antibiotic is shown here.
