# Supplementary material for: The Penicillin-Binding Protein PbpP Is a Sensor of β-Lactams and Is Required for Activation of the Extracytoplasmic Function σ Factor σP in Bacillus thuringiensis
Source: mBio. 2021 Mar 23;12(2):e00179-21. doi: 10.1128/mBio.00179-21 (PMC8092216; doi:10.1128/mBio.00179-21)
Supplement: FIG S8 [file mBio.00179-21-sf008.pdf]

Figure S8

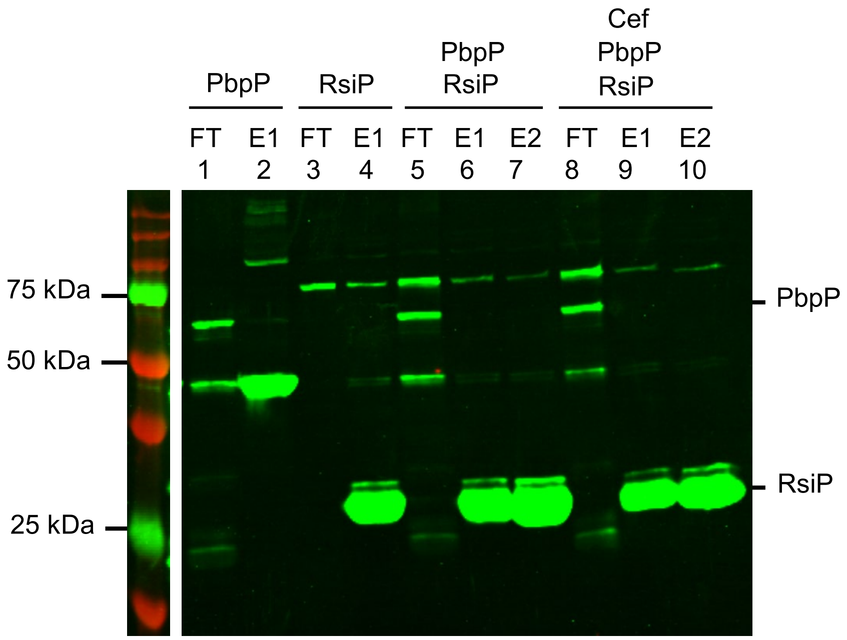

Figure S8. PbpP does not bind RsiP *in vitro.* 6xHis-RsiP was purified from *E. coli*. PbpP was produced *in vitro* using PURExpress *In Vitro* Protein Synthesis Kit and then run over a nickel column to eliminate 6xHis-tagged proteins. An aliquot of *in vitro* produced PbpP was incubated without RsiP (wells 1 and 2). 6xHis-RsiP alone is in wells 3 and 4. 6xHis-RsiP incubated with PbpP (wells 5-7). 6xHis-RsiP incubated with PbpP and cefoxitin (50 mg/mL) (wells 8-10). After incubated, the columns were washed and eluted. The unbound is annotated as flow through (FT) and elution fractions are designated with E1 or E2.
