# Supplementary material for: The Penicillin-Binding Protein PbpP Is a Sensor of β-Lactams and Is Required for Activation of the Extracytoplasmic Function σ Factor σP in Bacillus thuringiensis
Source: mBio. 2021 Mar 23;12(2):e00179-21. doi: 10.1128/mBio.00179-21 (PMC8092216; doi:10.1128/mBio.00179-21)
Supplement: TABLE S1 [file mBio.00179-21-st001.pdf]

TABLE S1. Plasmids used in this study

Table S1. Plasmids

Plasmid	Relevant features	Parent vector	Restriction enzymes to digest parent vector	PCR primers	PCR template	Reference
pMAD	ori-pE194ts , amp, erm					Arnaud 2004
pAH9	ori-pE194 P_{sarA} -*mcherry* , amp, erm					Malone 2009
pJAB980	ICE::P_{IPTG} -*gfp* *amp, cat*					Brophy et al 2018
pAC68	*thrC* ::P_{xyl} - *amp, erm*					Arnaud Chastanet
pDR111	*amyE* ::P_{IPTG}- *amp, spec*					David Rudner
pRAN332	P_{tet} -*gfp cat*					Ransom et al 2014
pEBT13	P_{tet} -*gfp-rsiP, amp, erm*					Ho et al 2019
pTHE950	pE194ts, *'thrC lacZ thrB'* , *cat*					Ho et al 2019
pTHE955	pE194ts, *'thrC* P_{pbpP} *-lacZ thrB'* , *cat*	pTHE950	XhoI, NotI	2933-2934	AW43	This study
pEBT2	ori-pE194ts, Δ*pbpP, amp, erm*	pMAD	BglII, EcoRI	3824-3825; 3826-3827	AW43	This study
pEBT10	ori-pE194 P_{pbpP} -*pbpP, amp, erm*	pAH9	HindIII, EcoRI	3849-3850	AW43	This study
pEBT20	ori-pE194 P_{tet} -*3488, amp, erm*	pAH9	HindIII, EcoRI	3838-3982; 3983-3850	pRAN332; AW43	This study
pCE693	ori-pE194 P_{tet} -*pbpP* ^{S301A} *amp, erm*	pAH9	HindIII, EcoRI	3838-3978; 3977-3850	pEBT20; pEBT20	This study
pCE785	ICE::P_{pbpP} -*pbpP* ^{S301A} *amp, cat*	pJAB980	PacI, NarI	4880-3978; 3977-4881	AW43	This study
pCE784	ICE::P_{pbpP} -*pbpP* *amp, cat*	pJAB980	PacI, NarI	4880-4881	AW43	This study
pCE707	ICE::P_{IPTG} -*pbpP* *amp, cat*	pJAB980	SalI, NheI	4531-4532	AW43	This study
pCE726	ICE::P_{IPTG} -*pbpP* ^{301A} *amp, cat*	pJAB980	SalI, NheI	4531-3978; 3977-4532	AW43	This study
pCE755	*thrC* ::P_{xyl} -*pbpP* *amp, erm*	pAC68	HindIII, BamHI	4743-4744	AW43	This study
pCE695	*amyE* ::P_{IPTG}-*gfp-rsiP* *amp, spec*	pDR111	HindIII, SphI	4564-4565	pEBT13	This study
pCE698	ICEBs1::P_{IPTG}-*gfp-rsiP* *amp, cat*	pJAB980	SalI, NheI	4515-4516	pEBT13	This study
pCE697	ICEBs1::P_{IPTG} *amp, cat*	pJAB980	PacI, NarI	4562-4563	AW43	This study
pCE593	P_{T7} -6xhis-*rsiP* $^{76-275}$ *amp*	pET21b-rtev	NcoI, EcoRI	3644-3643	AW43	This study
pCE830	P_{T7}-*pbpP* $^{35-586}$ *amp*	pET21b-rtev	NdeI, EcoRI	5170-4567	AW43	This study
