# Supplementary material for: The Penicillin-Binding Protein PbpP Is a Sensor of β-Lactams and Is Required for Activation of the Extracytoplasmic Function σ Factor σP in Bacillus thuringiensis
Source: mBio. 2021 Mar 23;12(2):e00179-21. doi: 10.1128/mBio.00179-21 (PMC8092216; doi:10.1128/mBio.00179-21)
Supplement: TABLE S2 [file mBio.00179-21-st002.pdf]

TABLE S2. Primers used in this study

Table S2. Primers

Oligo	Sequence	Relevant features
CDEP4880	tctttttcccgatgattaataaatcctccatttaatcctttcag	Clone P_{pbpP}-*pbpP* onto pCE697 digested w/ PacI and NarI
CDEP4881	ggcggtttgcgtattgggcgtcactttaatgaaccttcctct	Clone P_{pbpP}-*pbpP* onto pCE697 digested w/ PacI and NarI
CDEP3824	acacattaactagacagatcactacctaaaaagcaccgtg	Deltion *pbpP* pMAD EcoRI BglII
CDEP3825	gctacataagggagggttataccttcctcttttaccatatagaa	Deltion *pbpP* pMAD EcoRI BglII
CDEP3826	atatggtaaaagaggaaggtataaccctcccttatgtagcc	Deltion *pbpP* pMAD EcoRI BglII
CDEP3827	ctgcagaagcttctagaattacatcttttttaatgatttgtggtg	Deltion *pbpP* pMAD EcoRI BglII
CDEP3838	acgacggccagtgccaagctctagcataaaaataagaagcctgc	Ptet-GFP-rsiP onto pAH9 w/ HindIII EcoRI
CDEP3982	ctccatctttttgtatgtaacataaaattttctcctttactgcaggagc	Clone P_{tet}-*pbpP* onto pAH9 EcorI HindIII
CDEP3983	gctcctgcagtaaaggagaaaatttt atgttacatacaaaaagatggag	Clone P_{tet}-*pbpP* onto pAH9 EcorI HindIII
CDEP3849	acgacggccagtgccaagctaaatcctccatttaatcctttcag	*pbpP* onto pAH9 w/ HindIII EcoRI
CDEP3850	tatgaccatgattacgaatttcactttaatgaaccttcctct	*pbpP* onto pAH9 w/ HindIII EcoRI
CDEP3977	gtgagtggttttatagtcgcagccatatttacataagattc	Clone *pbpP*^{S301A}
CDEP3978	gaatcttatgtaaatatggctgcgactataaaaccactcac	Clone *pbpP*^{S301A}
CDEP4531	taacaattaagcttagtcgactacataagggagggttatatgt	Clone *pbpP* onto pJAB980 digested w/ SalI and NheI
CDEP4532	attagcttgcatgcggctagaagaggaaggttcactttaatg	Clone *pbpP* onto pJAB980 digested w/ SalI and NheI
CDEP4743	gacctgcaggcatgcaagctgatgaaataggctacataaggg	Clone BT3488 into pAC68 digested w/ HindIII BamHI
CDEP4744	aaaactgctgccttcggatctcactttaatgaaccttcctct	Clone BT3488 into pAC68 digested w/ HindIII BamHI
CDEP4564	ccaccgaattagcttgcatgatgaccatgattacgaattcc	clone GFP-RsiP onto pDR111 digested with HindIII SphI
CDEP4565	gagcggataacaattaagctagatctgagctcctgcagta	clone GFP-RsiP onto pDR111 digested with HindIII SphI
CDEP4515	taacaattaagcttagtcgaagatctgagctcctgcagta	Clone GFP-RsiP onto pJAB980 digested w/ SalI and NheI
CDEP4516	attagcttgcatgcggctagttaccatatagaatctatatttattattttcca	Clone GFP-RsiP onto pJAB980 digested w/ SalI and NheI
CDEP4562	tctttttcccgatgattaattaattcagaacgctcggttg	Drop out GFP from pJAB980
CDEP4563	attagcttgcatgcggctagctcaccacctttcagccgagtc	Drop out GFP from pJAB981
CDEP2933	gcaatgcccggtttttttctcgagaaatcctccatttaatcctttc	Clone P_{pbp}-*lacZ*
CDEP2934	tcaacaagctggggatccgcggccgcatataaccctcccttatgtag	Clone P_{pbp}-*lacZ*
CDEP3644	tcgacgtaggcctttgaattttaccatatagaatctatatttattattttcca	Clone 6xHis-RsiP$^{76\text{-}275}$ for T7 inducible expression
CDEP3643	tgtattttcagggcgccatggatgataataaatcaccggtacaaa	Clone 6xHis-RsiP$^{76\text{-}275}$ for T7 inducible expression
CDEP5170	ttaagaaggagatatacatatgacttcttcagctgaaagaggaa	Clone untagged PbpP$^{35\text{-}586}$ for T7 inducible expression
CDEP4567	tcgacgtaggcctttgaattatatagattctatatggtaaaagaggaag	Clone untagged PbpP$^{35\text{-}586}$ for T7 inducible expression

All sequences are 5' to 3'
